# Fatty acid synthase (FASN) signalome: A molecular guide for precision oncology

**DOI:** 10.1002/1878-0261.13582

**Published:** 2024-01-18

**Authors:** Javier A. Menendez, Elisabet Cuyàs, Jose Antonio Encinar, Travis Vander Steen, Sara Verdura, Àngela Llop‐Hernández, Júlia López, Eila Serrano‐Hervás, Sílvia Osuna, Begoña Martin‐Castillo, Ruth Lupu

**Affiliations:** ^1^ Metabolism & Cancer Group, Program Against Cancer Therapeutic Resistance (ProCURE) Catalan Institute of Oncology Girona Spain; ^2^ Girona Biomedical Research Institute Girona Spain; ^3^ Institute of Research, Development and Innovation in Biotechnology of Elche (IDiBE) and Molecular and Cell Biology Institute (IBMC) Miguel Hernández University (UMH) Elche Spain; ^4^ Division of Experimental Pathology, Department of Laboratory Medicine and Pathology Mayo Clinic Rochester MN USA; ^5^ Mayo Clinic Cancer Center Rochester MN USA; ^6^ Department of Biochemistry and Molecular Biology Laboratory Mayo Clinic Laboratory Rochester MN USA; ^7^ CompBioLab Group, Institut de Química Computacional i Catàlisi (IQCC) and Departament de Química Universitat de Girona Girona Spain; ^8^ ICREA Barcelona Spain; ^9^ Unit of Clinical Research Catalan Institute of Oncology Girona Spain

**Keywords:** cell fate, ferroptosis, immunotherapy, metastasis, mitochondrial priming, molecular glues

## Abstract

The initial excitement generated more than two decades ago by the discovery of drugs targeting fatty acid synthase (FASN)‐catalyzed *de novo* lipogenesis for cancer therapy was short‐lived. However, the advent of the first clinical‐grade FASN inhibitor (TVB‐2640; denifanstat), which is currently being studied in various phase II trials, and the exciting advances in understanding the FASN signalome are fueling a renewed interest in FASN‐targeted strategies for the treatment and prevention of cancer. Here, we provide a detailed overview of how FASN can drive phenotypic plasticity and cell fate decisions, mitochondrial regulation of cell death, immune escape and organ‐specific metastatic potential. We then present a variety of FASN‐targeted therapeutic approaches that address the major challenges facing FASN therapy. These include limitations of current FASN inhibitors and the lack of precision tools to maximize the therapeutic potential of FASN inhibitors in the clinic. Rethinking the role of FASN as a signal transducer in cancer pathogenesis may provide molecularly driven strategies to optimize FASN as a long‐awaited target for cancer therapeutics.

## Graphical abstract

This perspective explores the therapeutic relevance of emerging FASN hallmarks in oncology. We elucidate how FASN affects cancer cell plasticity and fate, controls mitochondrial cell death, helps cancer cells with immune evasion, and influences how and where cancer spreads. By understanding these novel FASN attributes, we propose a series of precision tactics for exploiting FASN in metabolic cancer therapy.

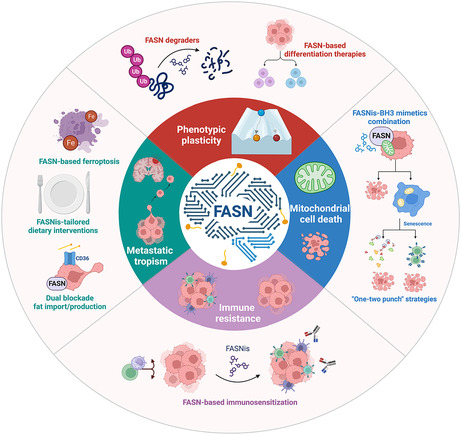

**Abbreviations**
ACCA: acetyl‐CoA carboxylase
ACIs: adoptive cell immunotherapies
ALDH: aldehyde dehydrogenase
APL: acute promyeolytic leukemia
ATRA: all‐trans retinoic acid
CAR‐T: chimeric antigen receptor T‐cell
CR: calorie restriction
CSC: cancer stem cells
CTLA‐4: cytotoxic T lymphocyte‐associated protein‐4
DBP: dynamic BH3 profiling
ECM: extracellular matrix
EMT: epithelial to mesenchymal transition
FASN: fatty acid synthase
FASTIS: fatty acid therapy‐induced senescence
IDH: isocitrate dehydrogenase
IFNγ: interferon gamma
iPSCs: induced pluripotent stem cells
KD: ketogenic diet
LFD: low‐fat diet
MOMP: mitochondrial outer membrane permeabilization
MUFAs: monounsaturated fatty acids
NF: nuclear factor
NK: natural killer
PD‐1: programmed cell death protein‐1
PD‐L1: programmed cell death‐ligand 1
POI: protein‐of‐interest
PROTACs: proteolysis‐targeted chimeras
PUFAs: polyunsaturated fatty acids
ROS: reactive oxygen species
SASP: senescence‐associated secretory phenotype
SCD: steaoryl‐CoA desaturase
SREBF1/SREBP1: sterol regulatory element binding transcription factor 1
TILs: tumor‐infiltrating lymphocytes
TME: tumor microenvironment
TPD: targeted protein degradation
T_reg_ cells: regulatory T cells

## 1 Introduction

**Fig. 1:**
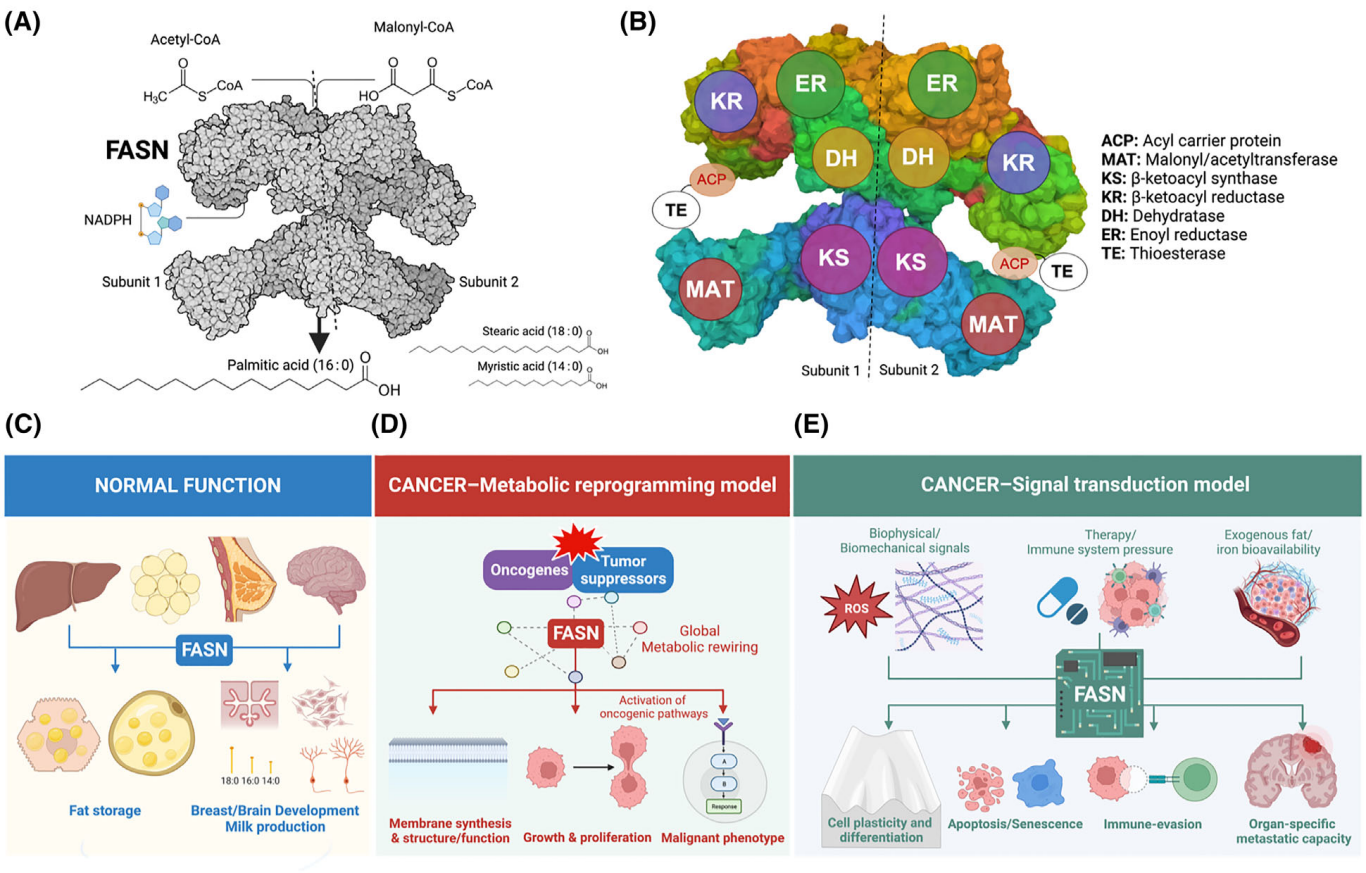
The megasynthase FASN: from catalysis to signaling. *Top panels*. Function and architecture of human FASN. (A) The megasynthase FASN is a multifunctional polypeptide enzyme that uses one acetyl‐CoA and seven malonyl‐CoA molecules to produce the 16‐carbon saturated FA palmitic acid (16:0). Although palmitic acid accounts for ~ 90% of the enzyme's product yield, FASN biosynthetic activity can also produce myristic acid (14 : 0) and stearic acid (18 : 0). (B) Schematic map of the FASN head‐to‐head homodimer model, with each monomer pair containing seven different active sites shown on the surface [1, 2]. ACP corresponds to the mobile acyl‐carrier protein domain that transports the substrate to the catalytic MAT, KS, KR, DH, ER and finally TE domains. Although the ACP domain is shown bound to KR, it binds all the other domains as it moves FA intermediates from one active site to another during the multi‐enzymatic cycle. The position of the TE domain, which is highly mobile, is for illustration only. The non‐catalytic methyltransferase and β‐ketoreductase‐like ‘pseudo‐domains’ of FASN have been omitted. *Bottom panels*. The biological dimensions of FASN in normal and cancerous tissues. (C) *Normal function*. FASN expression is relatively quiescent in most adult tissues, with the strongest expression occurring in the liver and adipose tissue, where it plays a critical role in metabolic homeostasis [3, 36, 37]. The specialized function of FASN as an endogenous source of short and medium‐chain FAs is essential – and cannot be substituted by extracellular FAs – for the functional development and maintenance of both the lactating mammary gland and the brain [4, 5]. (D) *Cancer–Metabolic reprogramming model*. Tumor FASN is part of the metabolic network rewiring programmed by common genetic and epigenetic alterations in oncogenes and tumor suppressor genes that occur in cancer cells [6, 7, 8, 9, 16]. In addition to configuring the architecture and function of the cancer cell membrane and playing a critical role in tumor growth and survival, FASN signaling also cross‐talks with other cancer signaling networks to generate and maintain the malignant phenotype. (E) *Cancer–Signal transduction model*. The modern view of tumor FASN is that of a signal transducer of biological information, in which FASN integrates the cellular state with environmental signals to execute cancer cell fate‐specific programs. For example, FASN can sense and respond to complex combinations of biomechanical/biophysical microenvironmental signals, therapy/immune system pressure or exogenous fat/iron bioavailability, and translate them into targeted programs to drive adaptive responses, including cell plasticity and differentiation, apoptotic cell death versus cell dormancy, immune‐evasion and organ‐specific metastatic capacity. Created with Biorender.com.

Fatty acid synthase (FASN) is the only enzyme that can endogenously synthesize long‐chain saturated fatty acids (FAs) *de novo* from acetyl‐CoA, malonyl‐CoA and reducing equivalents in the form of NADPH [1, 2] (Fig. 1A). The lipogenic activity of the FASN multi‐domain homodimer (Fig. 1B) is normally restricted to the liver and adipose tissue, where it converts excess carbohydrates into FAs, which are then esterified as triacylglycerol stores for later energy supply via β‐oxidation (Fig. 1C) [3]. Most normal cells acquire circulating lipids from the diet and from hepatic *de novo* FA synthesis. The exceptions to this are the lactating mammary gland and the brain. In these tissues, dietary fat cannot substitute for an obligatory physiological stimulation of FASN activity to enable development and functional competence (Fig. 1C) [4, 5]. Cancer cells, however, must secure an additional supply of lipids to fuel tumor development and progression [6, 7, 8, 9, 10]. Several oncogenes and (loss of) tumor‐suppressors co‐opt and decouple the metabolic output of FASN from its normal regulatory inputs, allowing cancer cells to generate endogenous lipid pools in a cell‐autonomous manner [11, 12, 13, 14, 15]. Accordingly, FASN‐driven *de novo* lipogenesis is one of the many anabolic processes reactivated to support robust and (abnormally) high levels of growth and proliferation in transformed cells (Fig. 1D). In 2007, we introduced the concept of a metabolo‐oncogenic nature of FASN to better understand its ability to interact with and regulate various cancer control networks, and its intrinsic capacity to drive malignant‐like phenotypes [16]. Such an input‐to‐output logic of the FASN signalome has become more evident in recent years with the recognition that FASN can respond to both endogenous and environmental inputs to direct cell fate conversion [17, 18, 19, 20, 21], cellular responses to stress [7, 22, 23, 24, 25, 26], immune escape [26, 27, 28, 29], and organ‐specific metastatic potential [10, 30, 31, 32, 33, 34, 35] (Fig. 1E).

**Fig. 2:**
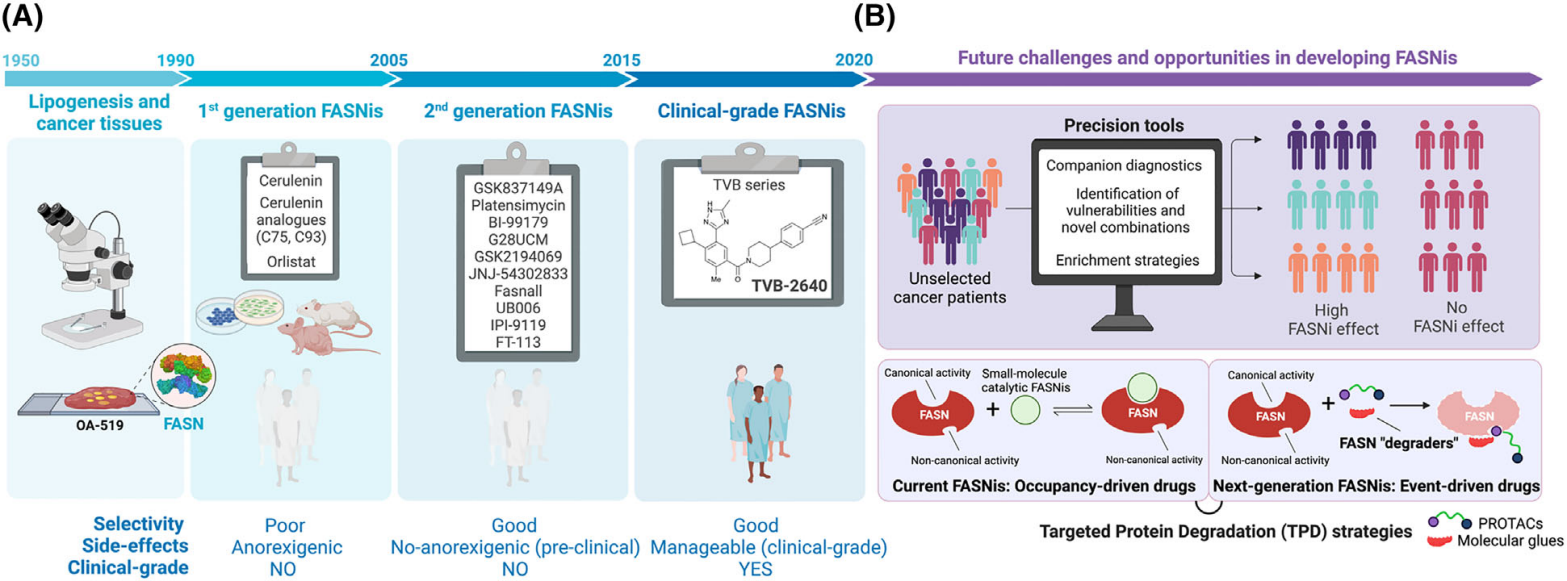
Key milestones in the timeline of FASN‐cancer discovery. (A) Associations between lipogenesis and cancer date back to the 1950s, when the ability of tumor tissue to convert glucose or acetate into FAs was first recognized [58]. In the mid‐1980s, it was demonstrated that cancer‐associated lipogenesis is almost exclusively derived from *de novo* FA synthesis rather than from preformed lipids in host tissues [59]. In the early 1990s, the obligatory requirement of some tumors for enhanced endogenous biogenesis of FAs – depending on the tumor type, cancer cells can synthesize up to 95% of saturated and monounsaturated FAs *de novo* despite adequate dietary lipid supply [60]) – was molecularly attributed to high levels of FASN expression and activity (formerly known as oncoantigen‐519) [61]. The almost universal overactivation of FASN in the majority of human carcinomas and their precursor lesions, together with the association of FASN overexpression with worse survival, suggested that FASN activity likely supports the high biosynthetic demands of cancer‐driven genetic lesions and confers metabolic advantages [62, 63, 64, 65]. Initial observations with naturally occurring FASNis such as the mycotoxin cerulenin, and shortly thereafter with synthetic cerulenin analogs (e.g. C75, C93, C247, CM‐55), confirmed that pharmacological blockade of FASN activity suppresses human cancer cell growth *in vitro* and reduces tumor burden in xenograft and genetically‐engineered mouse models [66, 67, 68, 69]. This first‐generation of FASNis failed to advance into the clinic due to unwanted reactivity and lack of specificity, but more importantly because they caused unmanageable side‐effects such as increased whole‐body energy expenditure and significant bodyweight loss [55, 56, 57]. Subsequent studies with more potent and selective FASNis (e.g. GSK837149A [70], platensimycin [71], BI‐99179 [72], G28UCM [73], GSK2194069 [74], JNJ‐54302833 [75], Fasnall [76], UB006 [77], IPI‐9119 [78], and FT‐113 [79]) strengthened the case for FASN as a target for cancer therapy, but this second generation of FASNis has remained in an early preclinical development despite having favorable pharmacological properties. The development of orally bioavailable, reversible, potent and selective next‐generation FASNis by 3‐V Biosciences (now Sagimet Biosciences) has demonstrated remarkable anti‐tumor potential of TVB FASNis in multiple preclinical models as well as improved tolerability and manageable systemic toxicity in early‐phase clinical trials [51, 52, 53, 54]. Importantly, TVB‐2640 – the most advanced clinical candidate – does not contribute to the indirect activation of peripheral carnitine palmitoyltransferase‐driven β‐oxidation, thereby avoiding the off‐target anorexigenic effects that were observed with first‐generation FASNis [55, 56, 57]. TVB‐2640 is currently being studied in several clinical settings, including *KRAS*‐mutant non‐small cell lung cancer (NCT03808558), resectable colorectal cancer (NCT02980029), relapsed high‐grade astrocytoma in combination with bevacizumab (NCT03938246), and HER2^+^ metastatic breast cancer resistant to trastuzumab and taxane‐based therapy (NCT03179904). (B) The predicted breakthrough of a new family of FASN‐targeted metabolic drugs will face three major challenges, namely: (1) the lack of companion or complementary diagnostics to aid in patient stratification and personalized therapeutic optimization of FASNis; (2) the lack of predictive enrichment strategies based on the pathomolecular mechanisms of cancer‐associated FASN; and (3) the lack of rational strategies to eliminate non‐catalytic functions that are insensitive to current FASNis. Created with Biorender.com.

Chemical FASN inhibitors (FASNis) were predicted in the 1990s to mitigate the malignant behavior of cancers. However, although drugs targeting other metabolic enzymes such as isocitrate dehydrogenase (IDH), glutaminase and arginase are already in – or *en route* – to clinical trials [38, 39, 40, 41, 42, 43], the initial excitement generated by the discovery of FASNis with anti‐tumor activity over three decades ago was short‐lived [44, 45, 46, 47, 48, 49, 50]. Pharmaceutical liabilities related to the efficacy, selectivity and safety of first‐ and second‐generation FASNis have been addressed by the development of reversible, 3‐V Bioscience (TVB) series of imidazopyridine‐based FASNis (TVB‐2640, TVB‐3166, TVB‐3664, TVB‐3693 and TVB‐3567) with optimized pharmacological properties, improved tolerability and superior target engagement and tumor responses (Fig. 2A) [51, 52, 53, 54, 55, 56, 57]. Although the progress of a clinical‐grade FASNi (TVB‐2640; denifanstat) now being studied in various phase II trials is stimulating renewed interest in FASN as a target for cancer treatment and prevention, there are several mechanistic weaknesses and therapeutic limitations that need to be addressed urgently (Fig. 2B). On the one hand, there remains a paucity of data to aid in the identification of the best precision tools and molecular enrichment strategies capable of maximizing the therapeutic potential of FASNis in the clinic. On the other hand, we are beginning to identify non‐catalytic, cancer‐promoting functions of FASN [23], which will require a shift from occupancy‐driven pharmacological approaches to event‐driven approaches involving the design of selective FASN protein ‘degraders’.

Here, we dissect the regulation and therapeutic relevance of emerging new dimensions of cancer biology involving FASN. First, we carefully review how canonical and non‐canonical FASN activities are involved in key molecular features of cancer pathogenesis such as unlocking phenotypic plasticity, mitochondrial regulation of cell death, immune escape and metastatic potential. Based on these new FASN hallmarks, we present and discuss new FASN‐centered therapeutic opportunities and clinical scenarios. These include the rational discovery of molecular glue degraders, the development of ‘one–two punch’ senogenic‐senolytic approaches, the modification of cancer cell susceptibility to immune cell‐mediated killing, and the synthetic lethal metabolic targeting of brain metastasis‐initiating cells. We propose to reconceptualize FASN as a critical signal transducer in cancer pathogenesis. This new perspective could lead to the development of more targeted, molecularly informed approaches to make FASN an effective and eagerly anticipated metabolic target in the field of precision oncology.

## 2 FASN: a driver of the ‘unlocking phenotypic plasticity’ cancer hallmark

**Fig. 3:**
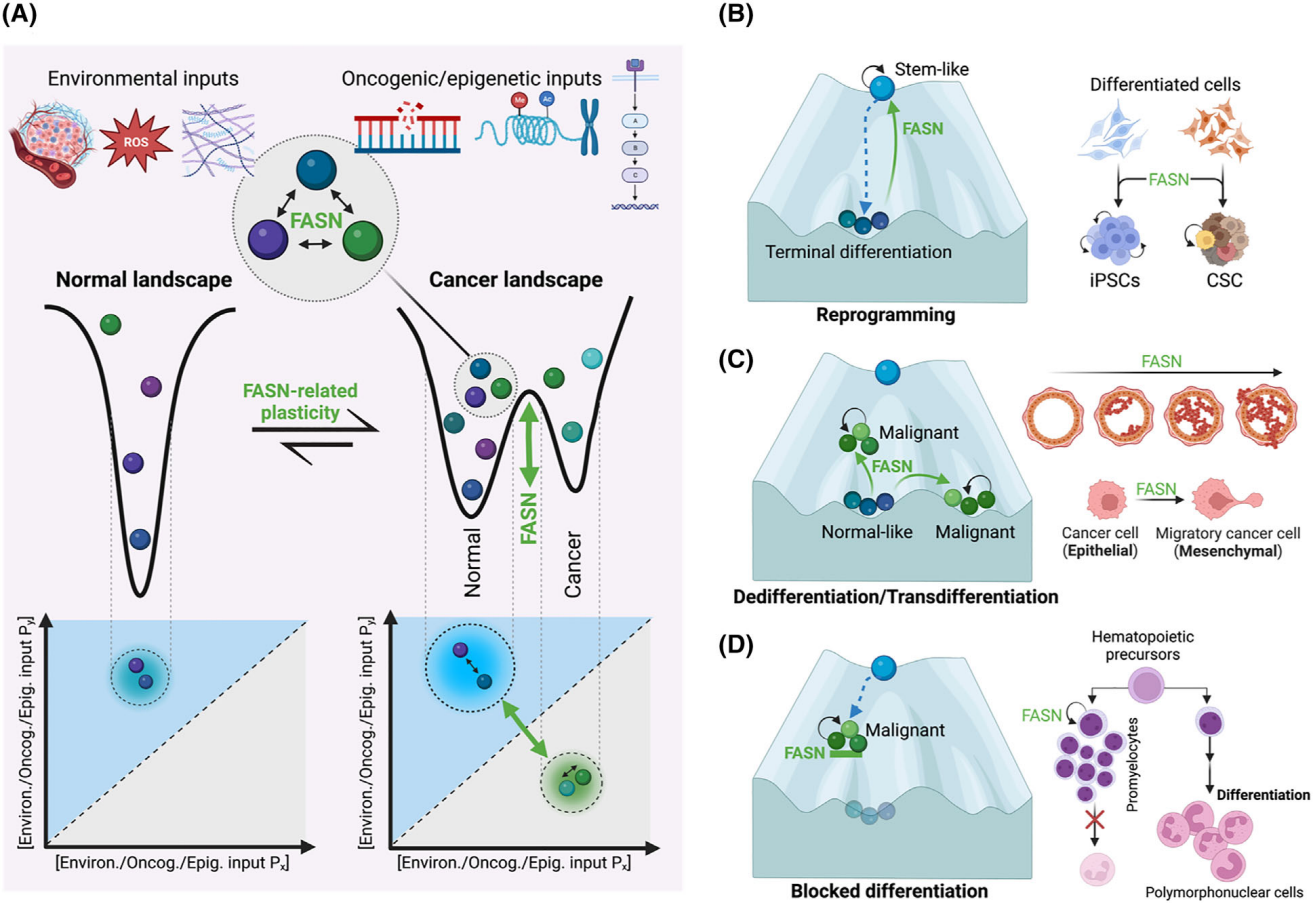
FASN: a mediator of plasticity in cancer pathogenesis. (A) FASN is a novel regulator of developmental pathways toward terminal differentiation in response to environmental, metabolic and oncogenic stresses. Accordingly, FASN can be considered part of regulatory networks that define the number and probability of stable cellular states (balls) that can be adopted by a population of cells, which represent attractors in the epigenetic landscape [83, 84, 85]. FASN signal may function as a landscape‐defining parameter (P) that can facilitate transitions (and corresponding phenotypic plasticity) between cellular states (balls) in response to various inputs (such as microenvironmental and oncogenic/epigenetic stimuli). FASN signaling may cooperate with the acquisition of aberrant developmental programs by altering the topography, transition rate and/or kinetic pathways of the epitranscriptional landscape. Activation of FASN signaling may be a common plasticity parameter of cell type switches (reprogramming, dedifferentiation and transdifferentiation) capable of remodeling the tumor tissue microenvironment during cancer initiation and progression. (B) FASN and FA synthesis are activated and are critical for the generation and maintenance of induced pluripotent stem cells (iPSCs) [18, 19, 21], a reprogramming phenomenon that involves reversion from a differentiated state to a pluripotent state with acquired unlimited proliferative properties and self‐renewal capacity. (C) By interfering with normal differentiation and promoting the erosion of barriers against dedifferentiation, FASN activation could play a permissive role in alleviating the developmentally unfavorable process of acquiring tumor‐initiating and/or metastasis‐initiating capabilities possessed by cancer stem cells (CSCs) [86, 87, 88]. By lowering the differentiation threshold that controls cell fate decisions in response to microenvironmental and oncogenic cues, FASN signaling may allow cells initially committed to one differentiation pathway to switch to another. (D) FASN may also be part of the regulatory changes that actively block the progression of incompletely differentiated progenitors with infinite self‐renewal to fully differentiated, typically non‐proliferative states. Further research is needed to reconstruct the structure of the cancer epigenetic landscape under different FASN‐driven metabolic conditions: FASN activation might exclusively facilitate cell state transitions without affecting the shape of the epigenomic landscape (e.g. via changes in FASN‐related epigenetic metabolites allowing for chromatin reorganization) and FASN activation could reshape the entire epigenomic landscape leading to new cell states (e.g. when a cell type has an indispensable FASN requirement or by increasing the entropy of the entire landscape to promote higher variability and occupancy of otherwise hidden attractors [89, 90]. Because unlocking the limited capacity for phenotypic plasticity to disrupt terminal differentiation facilitates the acquisition of other cancer hallmarks, targeted FASN suppression would represent a novel avenue for the development of differentiation‐inducing therapies in solid tumors. Created with Biorender.com.

Unlocking the limited capacity for phenotypic plasticity and proliferative capacity of terminally differentiated tissues is a recently recognized cancer hallmark [80]. Studies have shown that FASN activation is a non‐mutational, acquired metabolic trait that enables multiple modes of phenotypic plasticity and aberrant differentiation in response to environmental and oncogenic stresses [18, 19, 21, 81, 82]. Thus, FASN is emerging as an often‐overlooked biological molecule that may be indicative of, or even a driver of, the reversal or deformation of the developmental landscape during the initiation and progression of cancer (Fig. 3A).

### 2.1 FASN allows stemness reprogramming

Tumors rely on subpopulations of so‐called cancer stem cells (CSCs), which are endowed with stem‐like properties such as the ability to self‐renew and pluripotency [91, 92, 93, 94, 95]. CSCs are thought to be responsible for tumor initiation, recurrence and metastasis because they are less sensitive to therapy compared with non‐CSC tumor cells. CSCs can be derived from non‐tumorigenic cells that reacquire a stem cell‐like phenotype by undergoing processes similar to those of cellular reprogramming into induced pluripotent stem cells (iPSCs), i.e. the dedifferentiation of committed epithelial cells into stem‐like states with only a minimal core of stemness transcription factors (Oct‐4, Sox‐2, Klf4, c‐Myc). Accumulating evidence suggests that certain metabolic changes act as pivotal signaling events that render terminally differentiated somatic cells susceptible to the transcriptional circuit rewiring required for stemness acquisition and concomitant refractoriness to differentiation [86, 87, 96, 97]. FASN‐mediated activation of *de novo* FA synthesis is a distinct metabolic feature that, together with changes in the glucose and amino acid metabolism, facilitates cellular reprogramming and is critical for the acquisition and maintenance of the pluripotency of iPSCs (Fig. 3B) [18, 19, 21].

The fundamental mechanistic aspects by which FASN signaling may alter the molecular barriers present in cellular identity‐underlying ‘epigenetic landscapes’ – where cell fates are represented as attracting valleys resulting from a complex regulatory network – to allow differentiated cells to more easily (re)‐enter into stem‐like cellular states, remain unclear (Fig. 3A). However, a multifaceted contribution of FASN to the generation and maintenance of CSC phenotypes seems plausible (Fig. 3B). First, FASN activity may control cellular reprogramming and stem cell pluripotency through lipid products that drive the mitochondrial dynamic balance toward fission, a common driver of stemness in iPSCs and CSC [21, 98]. Secondly, activation of FASN may provide key metabolites that, by altering membrane properties and associated signaling pathway activities, may be indispensable for ensuring cell survival of stem‐like cell states. Accordingly, FASN inhibition efficiently and specifically eliminates the tumorigenic fate of the residual undifferentiated stem cells that, by functioning as *bona fide* tumor‐initiating CSCs, are responsible for the undesired teratocarcinoma growth of human iPSC derivatives [19]. Thirdly, FASN metabolic activity can function as a key upstream regulator of the epigenome. The enhanced production of lipid precursors upon activation of FASN‐centered *de novo* lipogenesis – a conserved transcriptional signature of naïve pluripotency [99] – includes epigenetically activating metabolites (e.g. alpha‐ketoglutaric acid and acetyl‐CoA). These metabolites may mediate changes in the global epigenetic landscape (e.g. DNA hypomethylation and histone hyperacetylation) that govern the generation and maintenance of this particular subpopulation of CSCs. Further research is urgently needed to investigate whether unscheduled FASN activation might affect the function of epigenetic modulators and modifiers in a feedback loop, thereby influencing lipid‐sensitive transcriptional circuits that are critical for the CSC phenotype [100]. Since only a few metabolic‐epigenetic axes appear to be compatible with the operational characteristics possessed by CSC cellular states, anti‐FASN drugs may target the molecular biology of cancer stemness itself. Accordingly, FASN inhibition reduces the expression of stemness markers in CSC while also reducing the ability of aldefluor (ALDH)‐positive CSCs to form mammospheres [68, 101, 102, 103, 104]. This could serve as an *in vitro* indicator of the content of tumor‐initiating CSC‐like cells in heterogeneous cancer cell populations.

### 2.2 FASN facilitates oncogenic dedifferentiation and transdifferentiation

*FASN* knockdown, but not the pharmacological blockade of its biosynthetic activity, can drive ‘phenotypic reversion’. This means that FASN can stably switch the undifferentiated, invasive phenotype of oncogenically transformed, metastatic breast cancer tissue back to a non‐malignant, normal‐like architecture. This reversal is nearly indistinguishable from that of the original breast tissue, including loss of proliferative potential and restoration of cell polarity and lineage markers [81]. Extracellular matrix (ECM)‐derived signaling has traditionally been considered as the ultimate regulator of the structure–function stability in normal tissue architecture [105, 106, 107, 108]. Conversely, the acquisition of independence from microenvironmental constraints is a critical feature of more aggressive tumorigenesis [109]. How then do we explain the dominant ability of *FASN* knockdown to dictate the organizational fate and phenotype of epithelial tissues over the presence of cancer‐driving genomic abnormalities? The contractile dynamics and the mechanical forces exerted by the ECM have recently been identified as potent inducers of transcriptional programs that culminate in acquiring a FASN‐positive lipogenic phenotype [110, 111]. FASN may therefore play a role in the physical dynamics that govern plasticity processes in migratory and invasive cancer cells (Fig. 3C). FASN activation may sensitize cells to intrinsic and microenvironmental oncogenic cues (e.g. genetic mutations, chromosomal aberrations, epigenetic changes, hypoxia, reactive oxygen species (ROS), nutrient deprivation, ECM structural configuration, chronic inflammation, immune system dysregulation, etc.) by lowering the threshold above which cell state transitions occur rather than directly promoting phenotypic transitions.

FASN signaling may function as a critical component of the dynamic, bidirectional signaling between cancer cells and their microenvironment that controls and regulates oncogenic dedifferentiation [112, 113]. This may explain why the sole elimination of the FASN protein mimics the ability of microenvironmental cues to suppress and reverse the three‐dimensional (3D) malignant phenotype of tumor cells irrespective of their genetic background [81]. In addition, FASN activation appears to contribute to the transdifferentiation of (static) epithelial cells into (mobile) mesenchymal cells [81, 114, 115, 116], a well‐described pathogenic event in cancer invasion, metastasis, and drug resistance. One intriguing possibility is that FASN signaling ultimately determines how ‘ready’ a cell is to alter its biophysical and/or biochemical properties in response to stimuli that can induce a non‐linear hysteretic epithelial‐to‐mesenchymal transition (EMT) [117, 118], a pro‐metastatic EMT mode in which cells not only acquire morphological changes and invasive potential, but also express subsets of stem cell and ECM‐related genes.

### 2.3 FASN activation reinforces differentiation blocks

Acute promyelocytic leukemia (APL) is the archetype of another type of phenotypic plasticity in which incompletely differentiated (myeloid) progenitors undergo regulatory changes that block their differentiation into non‐proliferative, terminally differentiated granulocytes [119, 120]. Indeed, the therapeutic paradigm of the ‘phenotypic reversion’ approach is the successful use of the RARα ligand all‐*trans* retinoic acid (ATRA) to inhibit the proliferation of abnormal blast cells and promote their differentiation into mature white blood cells [121, 122]. More widespread use of ATRA has been largely unsuccessful due to its inability to induce a differentiation phenotype in non‐APL neoplastic cells. Reducing FASN protein levels – but not inhibiting its activity with FASNis– can accelerate the differentiation of APL cell lines and re‐sensitize non‐APL neoplastic cells to differentiate into cells with a more mature phenotype in the presence of ATRA [82]. The non‐catalytic role of FASN in the differentiation of immature acute myeloid leukemia blasts involves a lysosome‐associated pool of FASN capable of activating the mTOR/TFEB autophagy‐lysosomal pathway [82]. In ATRA‐refractory neuroblastoma – an embryonal malignancy that arises from aberrant or blocked differentiation of neural crest‐derived multipotent stem cells [123, 124, 125, 126] – FASNis have been shown to promote neural maturation and differentiation [20]. In the scenario where the *MYCN*‐oncogene drives pluripotency and impedes the spontaneous differentiation of high‐risk neuroblastomas [127, 128, 129, 130], FASN blockade recouples and redirects neuroblastoma cells to terminal differentiation via activation of ERK signaling [20]. The activation of FASN is a metabolic feature that appears to reinforce the blockage of developmental pathways toward terminal differentiation in progenitor/stem cell‐like states that are imposed by a variety of oncogenic aberrations (Fig. 3D).

### 2.4 FASN protein degraders: a novel class of differentiation‐inducing therapeutics yet to be developed

Manipulation of FASN signaling to phenotypically override the unstable cancer genome in neoplastic cells is analogous to the results from differentiation‐inducing therapy, which aims to convert undifferentiated cancer cells into differentiated cells with low tumorigenicity [120, 121, 131, 132]. However, the observation that the non‐catalytic functions of FASN, but not necessarily its biosynthetic activity, control the functional specialization in cell‐fate decisions underscores the limitations of using traditional, occupancy‐driven small‐molecule FASNis for differentiation therapies. In fact, the obligatory requirement for FASN activation during the 2D‐to‐3D growth transition in the transformation step of mammary tumorigenesis is completely unrelated to its enzymatic product. Rather, it is due to its ability to unlock IDH1‐dependent reductive carboxylation to produce reactive oxygen species (ROS)‐quenching reducing equivalents [23, 24]. This non‐catalytic, palmitate‐independent regulatory activity of FASN on early metabolic reprogramming of malignant cells may explain, at least in part, why the tumor efficacy of most FASNis in established primary tumors is transient. An elegant way to target simultaneously the non‐catalytic and catalytic functions of FASN would be to develop event‐driven pharmacological approaches via targeted protein degradation strategies [133, 134, 135, 136, 137, 138, 139, 140].

In contrast to the currently available FASNis, which block enzymatic activity, FASN degraders such as proteolysis‐targeting chimeras (PROTACs) and molecular glues are expected to hijack the mechanisms that cells use to endogenously destroy FASN. PROTACs are heterobifunctional small‐molecule degraders consisting of a ligand that binds a protein of interest (POI), the so‐called ‘warhead’, a linker, and a ligand for an E3 ubiquitin ligase. Unlike PROTACs, small molecule glue‐like compounds are monovalent degraders that bind both the target protein and a substrate receptor within an E3 ubiquitin ligase by embedding into and stabilizing a naturally occurring protein–protein interaction (PPI). In both cases, the bridging between a POI (e.g. FASN) and the E3 ligase mediated by the degraders allows direct and cooperative binding between the protein pair, facilitating polyubiquitination and proteasomal degradation, ultimately leading to the suppression of a POI‐driven cellular response. The unique mechanism of action of PROTACs and molecular glues, which can degrade POIs in a sub‐stoichiometric and catalytic manner, provides a new means to eliminate the non‐catalytic functions of FASN [22, 81, 82]. Additionally, this approach may also circumvent the commonly overlooked problem of effective dosing of intracellular metabolic enzymes such as FASN. Physiologically, cancer‐associated overexpressed FASN reaches protein levels in the two‐ to three‐digit μm range (e.g. up to 28% of cytosolic protein weight is constituted by FASN in the breast cancer cell line SKBR3 [61]). These intracellular concentrations of FASN would require equally high concentrations of FASNis to ensure the complete neutralization of activity, which could pose pharmacokinetic problems.

Although PROTAC technologies may overcome the limitations of occupancy‐driven FASNis, including the need for continuous drug exposure, the development of FASN PROTACs is limited by the lack of FASN‐binding ligands. However, given that FASN is a natural substrate for E3 ubiquitin‐ligases including COP1 [141] and TRIM21 [142, 143, 144, 145], which are known to promote the polyubiquitination and proteasomal degradation of FASN, the discovery of FASN‐targeted molecular glues could be rationally explored. The prospective discovery of potent molecular glues that can not only repair but also potently enhance the lost binding of an oncogenic mutant protein to its cognate E3 ligase, thereby restoring its ubiquitination and proteasomal degradation [146], has demonstrated the feasibility of this targeted approach. A similar structure‐based approach aimed at enhancing the interactions of FASN with a native substrate of its cognate E3 ligases (rather than hijacking neosubstrates) could drive the rational discovery and optimization of molecular glue‐like FASN degraders.

#### 2.4.1 Rational discovery of FASN‐degrading molecular glues: exploiting the interaction of FASN with its cognate E3 ligases

**Fig. 4:**
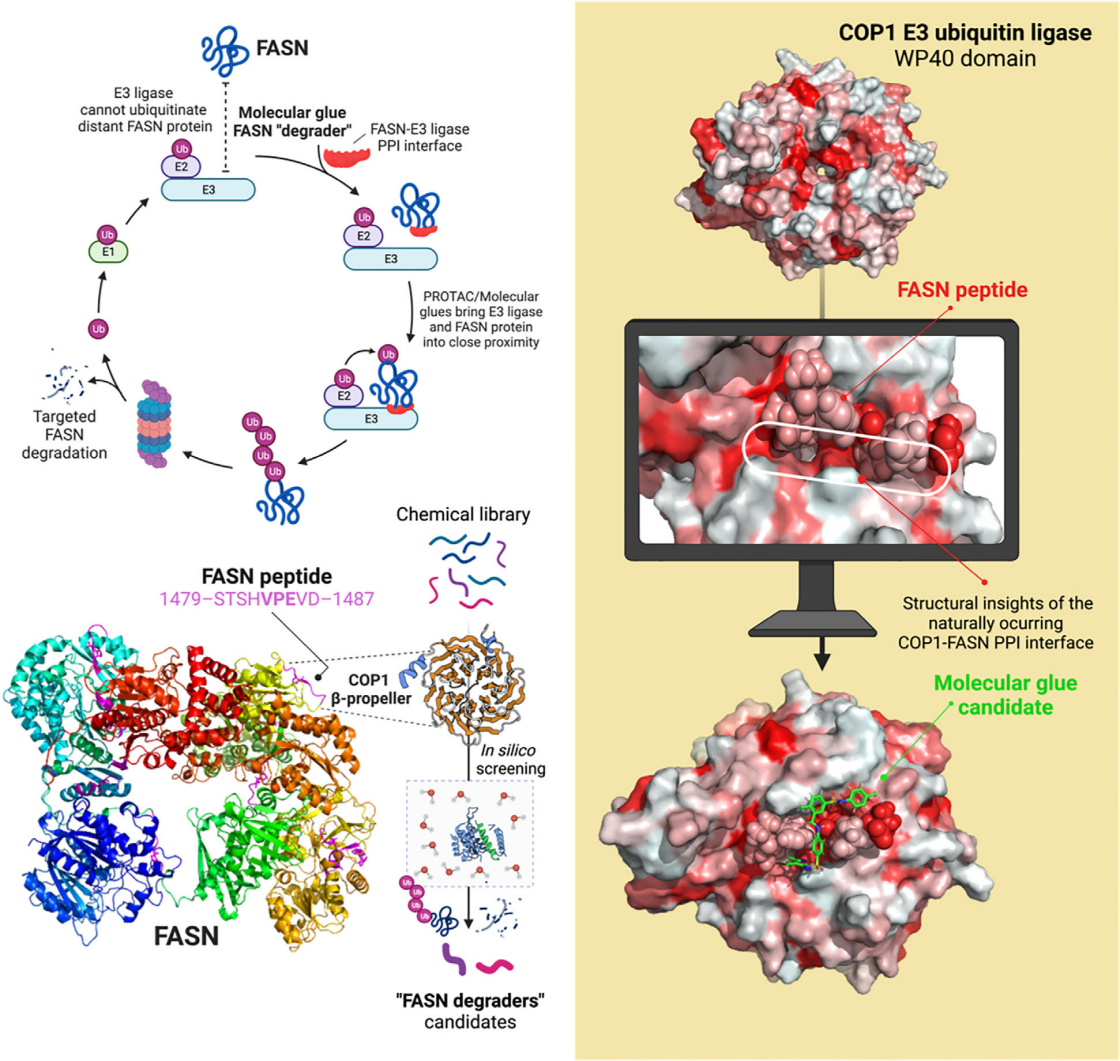
Development of selective drug‐like molecular glues for the protein–protein interaction FASN/COP1. Unlike PROTACs, which can achieve warhead degradation selectivity through specificity within the ternary E3 ligase complex, molecular glues must be inherently specific for the PPI complex that they stabilize. Careful study of the crystal structure of the COP1‐FASN binary complexes may identify druggable pockets at the corresponding PPI interfaces, which can guide the development of *high‐throughput screening platforms* specifically designed to identify molecular glue‐like FASN degraders. These compounds could be the first chemical tools to study non‐canonical FASN activity and inform future FASN modulators with a broad applicability, while circumventing the problems of classical FASNis interacting with one or more FASN catalytic domains. Created with Biorender.com.

COP1 is a key regulator of cell metabolism, including glucose homeostasis by degrading phosphorylated TORC2 [147], and lipid biogenesis by promoting the degradative polyubiquitination of the key lipogenic enzymes FASN and acetyl‐coenzyme A carboxylase (ACCA) [141, 148, 149]. COP1 is a single‐subunit RING‐type ligase that contains a ligandable, substrate‐binding region within the so‐called WD40 repeat domain [150]. Identification of the structural basis and key interactions of the target motif recognition by COP1 has revealed a universal mechanism involving the consensus VPE sequence [151, 152]. VPE interacts with a defined surface area of the β‐propeller motif of the COP1 WD40 repeat domain through both hydrophobic and ionic interactions, allowing substrate recognition and targeting for ubiquitination. It should be investigated whether the human FASN sequence contains VPE motifs or degrons as extended and outer sequences of the protein, which could make them ideal COP1‐FASN interacting regions for the discovery and development of drug‐like FASN degraders (Fig. 4). The availability of such structural information could enable high‐throughput computational screens using molecular docking and molecular dynamics simulations [153] to search for small molecules capable of ‘locking’ FASN to COP1 and increasing the rate of FASN degradation.

FASN is one of the several metabolic enzymes that have been identified as substrates of TRIM21, a RING finger domain‐containing E3 ubiquitin ligase belonging to the family of the tripartite motif (TRIM) family [154]. FASN acetylation catalyzed by the lysine acetyltransferase KAT8 destabilizes the FASN protein by promoting its interaction with TRIM21, resulting in inhibition of endogenous FA synthesis [142]. Glyceronephosphate O‐acyltransferase (GNPAT), a rate‐limiting enzyme in the biosynthesis of a special class of ether‐phospholipids called plasmogens [155, 156], is another TRIM21‐related stabilizer of FASN [143]. In response to exogenous stimulation with palmitic acid, acetyl‐CoA acetyltransferase 1 (ACAT1) acetylates GNPAT, which can competitively repress TRIM21‐mediated degradation of both GNPAT and FASN to promote tumorigenic lipid metabolism [143]. Glucose deprivation favors the interaction of phosphorylated FASN with TRIM21 for FASN degradation. Conversely, high glucose‐induced neddylation of PTEN leads to FASN dephosphorylation, thereby preventing TRIM21 from interacting with FASN to promote *de novo* FA synthesis [144, 145]. TRIM E3 ubiquitin ligases bind their substrates via the C‐terminal PRY/SPRY domain, a major autoantigen in autoimmune diseases that has been resolved in complex with the Fc region of the immunoglobulin IgG, revealing a well‐defined pocked at the IgG binding site [157, 158]. The residues within the TRIM21 PRY/SPRY domain that cluster in the center of the interface and interact with IgG Fc may also be critical for TRIM21 interaction with specific FASN sites, which can be modeled in the TRIM21 cavity to allow the subsequent identification and optimization of compounds with glue‐like activity.

Targeted FASN protein degradation using PROTAC/molecular glue technologies or FASN deubiquitinase inhibitors could be the cornerstone of a new family of differentiation‐inducing drugs for solid tumors. With more drug‐like properties than PROTACs, including a lower molecular weight, improved cellular permeability and better oral bioavailability, a search for molecular glues is warranted to accelerate the discovery of clinically valuable FASN degraders. Alternatively, ubiquitin‐specific cysteine protease 2a (USP2a), a deubiquitinating enzyme that rescues FASN from proteasomal degradation and mediates its stabilization [159, 160], could be exploited. Inhibitors of USP2a [161, 162, 163, 164] are predicted to promote FASN protein degradation, suggesting that selective small‐molecule inhibitors of USP2a would phenocopy the function of PROTACs/molecular glues as *bona fide* FASN degraders.

## 3 FASN: a novel regulator of mitochondrial cell death

**Fig. 5:**
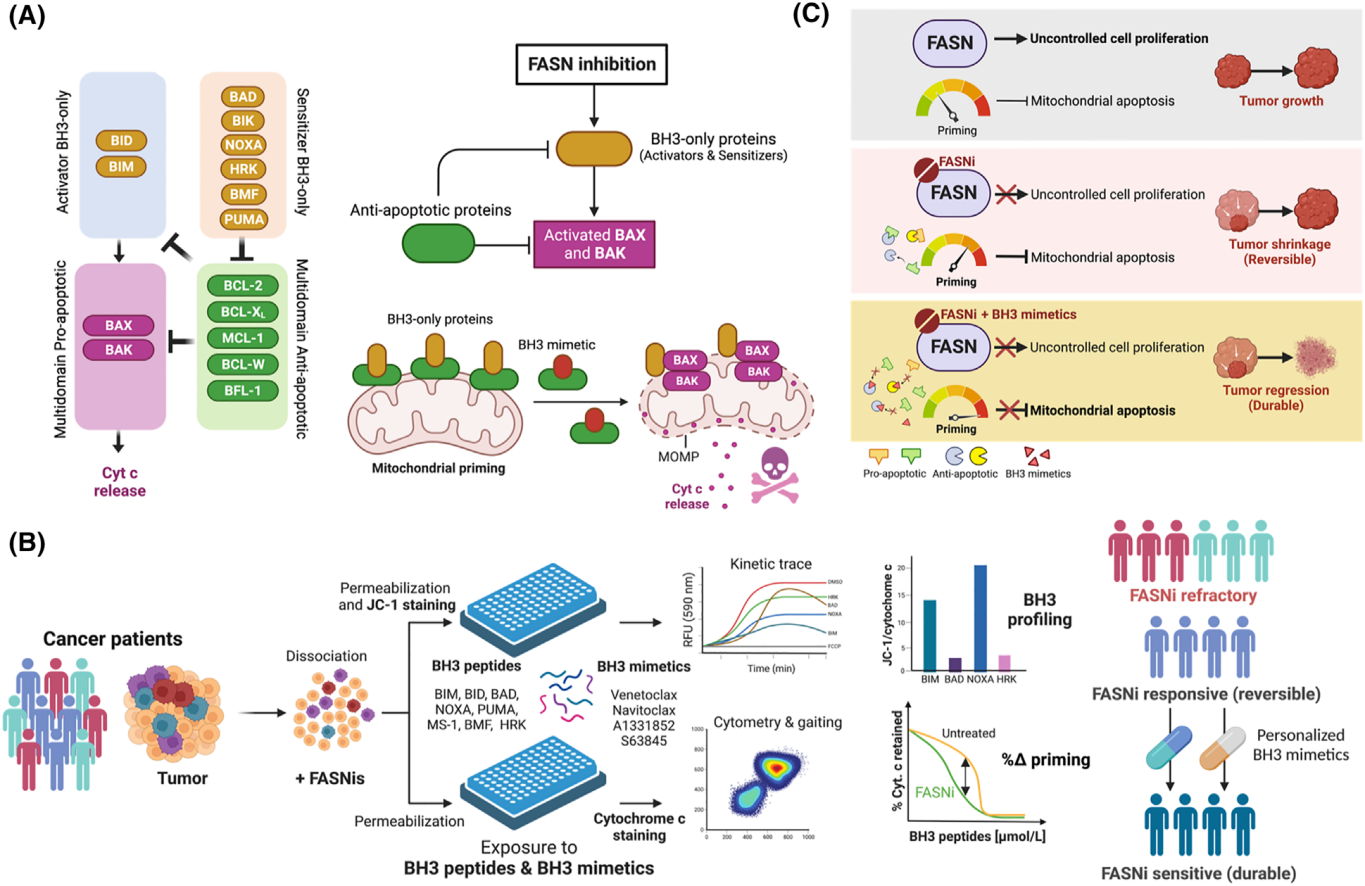
FASN and mitochondrial apoptotic cell death: from molecular mechanisms to personalized use of FASNis. (A) FASN regulation of the BCL‐2 interactome dictates therapeutic responses to FASNis. The execution of apoptosis requires the effector members BAX and BAK, but the threshold required for their activation is determined by the dynamic binding and expression of other subsets of the BCL2‐family of proteins, including activator and anti‐apoptotic members, as well as sensitizers [175, 176, 177, 178, 179, 180]. The activator proteins BID, BIM and PUMA, each possessing a BH3 domain, can directly interact with BAX and BAK to induce their homo‐oligomerization and MOMP, leading to apoptosis. The anti‐apoptotic proteins BCL‐2, BCL‐X_L_, MCL‐1, BCL‐W and BFL‐1/A1 prevent apoptosis by sequestering and neutralizing the BH3 domains of activators or effectors. The sensitizer proteins BAD, HRK, BIK, BMF and NOXA, which also possess a BH3 domain, cannot directly activate BAX and BAK but instead exert their pro‐apoptotic function by competing for the BH3 binding site in the anti‐apoptotic members, displacing the bound effectors and activators. Cancer cell death occurs when the sequestration capacity of anti‐apoptotic proteins is exceeded by levels of pro‐apoptotic proteins, which can be achieved pharmacologically with so‐called BH3 mimetics, which are able to interfere with the ability of anti‐apoptotic proteins to neutralize activating events driven by BH3‐only proteins. FASNis put FASN‐positive cancer cells into a primed‐for‐death mitochondrial state that relies on anti‐apoptotic BCL‐2 proteins to ensure survival, thereby allowing a BH3‐mimetic BCL‐2 inhibitor to unleash the mitochondrial apoptotic pathway [26]. Accordingly, counteracting the binding of pro‐apoptotic proteins with either the pan‐BCL inhibitor navitoclax or the BCL2‐specific inhibitor venetoclax abolishes this protected state, leading to enhanced apoptosis in FASN‐inhibited cancer cells [26, 181]. (B) BH3 profiling: an opportunity for functional precision medicine using FASNis. Using BH3 profiling and its variant, dynamic BH3 profiling (DBP), both of which involve the exposure of cellular mitochondria to synthetic ~ 20‐mer pro‐apoptotic BH3 peptides that mimic the activity of endogenous pro‐apoptotic proteins to induce MOMP [175, 176, 177, 178, 182, 183, 184, 185, 186], could be used to predict and monitor patient response to FASNis. Using either activator or sensitizer peptides, BH3 profiling can determine both the level of overall mitochondrial apoptotic priming – a general indicator of increased sensitivity to FASNis – and the specific survival dependencies on anti‐apoptotic proteins adaptively activated in response to FASNis. Priming‐based prospective identification of patients who are unlikely to respond to FASNis could spare them from ineffective and potentially toxic regimens, while at the same time helping to rationally assign individual patients to the most beneficial combination of FASNi and BH3 mimetics. (C). Mitochondrial apoptotic priming: unlocking the full therapeutic potential of FASNis. Although they prevent FASN‐driven cell proliferation, most FASNis have cytostatic effects that fail to eliminate tumors and are rapidly reversible. This is because FASN‐inhibited cancer cells can ensure survival with increased mitochondrial priming by becoming ‘addicted’ to anti‐apoptotic proteins to sequester pro‐apoptotic BH3‐only proteins and prevent mitochondrial apoptotic cell death (see A). BH3 mimetics can trigger the intrinsic apoptotic pathway in FASN‐inhibited cancer cells primed‐for‐death by binding to pro‐survival proteins and releasing activator proteins [187, 188, 189]. This model reveals a critical vulnerability in the regulation of the mitochondrial apoptotic machinery [190, 191] downstream of FASN signaling that, if translatable to the clinic, could convert the reversible tumor shrinkage in response to FASNi monotherapy into a more complete/durable tumor regression in combination with BH3 mimetics. Created with Biorender.com.

The molecular determinants of cancer cell sensitivity to FASNis are largely unknown, which is a major obstacle in their therapeutic application. The efficacy and durability of anti‐cancer FASNis will likely depend on their ability to target the cell death pathways that are commonly dysregulated in tumors [165, 166, 167, 168, 169]. Therefore, understanding how cancer cells die in response to FASNis or how cancer cells evade cell death in response to FASNis, has the potential to improve cancer responses to FASNis. Some studies initially suggested that the intracellular accumulation of malonyl‐CoA and ceramide upon FASN blockade triggered the extrinsic apoptotic pathway [170]. However, the majority of studies have proposed that FASNis instead activate the intrinsic mitochondrial pathway in both cancerous and non‐cancerous cell lines, as evidenced by cytochrome c release and caspase activation following increased ROS production [171, 172, 173, 174]. We have recently described that the ability of FASN signaling to dictate the commitment to mitochondrial cell death by the BCL‐2 family of proteins, contributes critically to the differential response to FASNis in cancer cells [26] (Fig. 5A).

### 3.1 FASN signaling regulates the mitochondrial primed‐for‐death state

The extent of apoptotic cell death in response to FASNis in cancer models closely reflects the baseline level of FASN expression. However, early apoptosis‐related events such as mitochondrial depolarization, which attenuates ROS generation, are a common adaptive response to FASNis that occurs regardless of FASN expression status [26]. Accordingly, FASNi‐driven redox stress induces metabolic adaptations that favor increased NADPH synthesis and decreased NADH production, and is accompanied by the activation of redox‐sensing kinases such as p38 MAPK and AMPK [26, 173]. FASNis promote mitochondrial outer membrane permeabilization (MOMP) and cytochrome c release only when the accumulated exposure to ROS leads to mitochondrial oxidative stress above a certain threshold. The point of commitment for apoptotic cell death in response to FASNis is ultimately determined by the tightly regulated interactions that occur within the so‐called BCL‐2 interactome (Fig. 5A).

Most anti‐cancer agents induce apoptosis by affecting the BCL2 interactome through the accumulation of pro‐apoptotic BH3‐only proteins (mostly activators, but also sensitizers), by decreasing the abundance of anti‐apoptotic BCL2 members, or both. In low and moderate FASN‐expressing cancer cells, the mitochondrial response to FASNis does not involve significant changes in the ratio of pro‐apoptotic and anti‐apoptotic signals and is therefore insufficient to cross the apoptotic threshold [26]. In the case of FASN‐overexpressing cancer cells, FASNis do not alter the expression status of pro‐apoptotic proteins, but instead promote the increased transcription and translation of pro‐death, BH3‐only activators (e.g. BIM) and sensitizers (e.g. PUMA and NOXA) [26], whose activation commits cells to apoptosis [175, 176, 177, 178, 179, 180]. However, the increased binding of pro‐apoptotic proteins to anti‐apoptotic BCL‐2 proteins prevents cell death in a significant number of FASN‐overexpressing cancer cells. Thus, although some FASN‐positive cancer cells are killed during treatment with FASNis, others survive. This phenomenon of killing across a threshold may be due to stochastic variations from cell to cell in the level and timing of activation of the BCL‐2 proteins that regulate apoptosis [192]. Pharmacological blockade of FASN activity enhances mitochondrial apoptotic priming by shifting FASN‐positive cancer cells to a higher state of death *readiness* that is dependent on certain anti‐apoptotic proteins to sequester BH3‐only activators/sensitizers and ensure survival (Fig. 5A). Accordingly, FASN loss‐of‐function leading to defective *de novo* FA synthesis [193] can significantly increase mitochondrial sensitivity to the synthetic BIM BH3 peptide [26], which binds promiscuously to all anti‐apoptotic BCL‐2 family members and is a proxy for general mitochondrial priming. Conversely, the up‐regulation of pro‐apoptotic BH3‐only proteins such as BIM by FASN inhibition can be rescued by the FASN end‐product palmitate [26]. This demonstrates that starvation of endogenously produced FAs is a previously unrecognized metabolic stressor that, similar to the starvation of extracellular glucose or amino acids [194, 195, 196, 197, 198], results in increased mitochondrial priming.

### 3.2 Mitochondrial apoptotic priming: from companion diagnostics to therapeutic personalization of FASNis

In the absence of genomic variations or protein aberrations driving the metabolo‐oncogenic properties of FASN, it remains a challenge to identify and select the best patients who could benefit from FASNis. The extracellular/circulating form of FASN (cFASN) has been is associated with active extrusion of intracellular FASN in metabolically demanding human diseases including cancer [199, 200, 201, 202, 203]. Elevated pretreatment serum levels of cFASN were found to correlate with response to TVB‐2640, supporting a putative role of cFASN in enriching for responders to FASNi‐based therapies [53]. With this exception, studies that provide immediately translatable, personalized information to guide FASNi‐based therapy are at the earliest stages of discovery. As noted above, we have recently learned that the level of mitochondrial priming correlates with, and may be a determinant of, the differential responsiveness of cancer cells to FASNis [26]. This scenario opens the door to incorporate functional precision oncology approaches such as BH3 profiling or its variant, dynamic BH3 profiling (DBP) [175, 176, 177, 178, 182, 183, 184, 185, 186] as companion diagnostics to distinguish responders from non‐responders in upcoming clinical trials with FASNis based on FASNi‐induced changes in mitochondrial priming without the need for classical genetic biomarkers [204, 205, 206, 207, 208] (Fig. 5B).

BH3 and DBP profiling assays may also enable the incorporation of BH3 mimetics for personalized combinatorial strategies that can be implemented depending on the specific pro‐survival proteins activated in response to FASNis. BH3 mimetics are small synthetic molecules that mimic the antagonistic function of specific pro‐apoptotic BH3‐only proteins to bind and sequester anti‐apoptotic BCL‐2 guardians. In doing so, they increase the apparent stoichiometry of BH3‐only proteins and shift the balance toward apoptosis in cells dependent on these pro‐survival proteins [178, 179, 180, 181, 187, 188, 189]. Treatment with the BH3 mimetics ABT‐263/navitoclax (which disrupts BCL‐2 and BCL‐X_L_ interactions) and ABT‐199/venetoclax (which exclusively antagonizes BCL‐2), was found to abolish the protected state in surviving cells [26]. However, this effect was not replicated with S63845 (antagonizing MCL‐1) or A1331852 (antagonizing BCL‐X_L_), Therefore, counteracting the binding of pro‐death proteins allows apoptotic cell death in FASN‐inhibited cancer cells *in vitro* and also a synergistic tumor‐killing effect in breast cancer xenograft models (Fig. 5B). Unlocking a potent anti‐tumor potential by combining FASNis with direct apoptosis activators seems to be a universal mechanism that can be applied even in difficult‐to‐treat tumors such as pancreatic cancer. In our hands, the combination of FASNis with BCL‐2is synergistically inhibited tumor growth and promoted apoptotic cell death in gemcitabine‐resistant, pancreatic cancer patient‐derived xenografts (PDX) and PDX‐derived cell lines (unpublished observations).

The activation status of FASN regulates the BCL‐2 interactome, mechanistically linking the redox‐buffering mechanism of FASN activity to the intrinsic apoptotic threshold in cancer cells. The survival versus death fate of cancer cells exposed to FASNis may depend on how their effect on the reorganization of the BCL‐2 network moves cancer cells toward the mitochondrial apoptotic threshold. The discovery that starvation of FASN‐produced endogenous FAs is a metabolic stress that enhances mitochondrial apoptotic priming may be a powerful means to rationally improve next‐generation FASNis in combination with BH3‐mimetic drugs. BH3 and DBP profiling assays can be used to establish definitively whether BCL‐2 is a pan‐cancer pro‐survival protein that dictates therapeutic responses to FASNis across cancer types (Fig. 5B). The exploration of combinatorial strategies involving FASNis and BH3 mimetics could hold promise for novel regimens that could achieve more complete and durable remissions in FASN‐addicted tumors (Fig. 5C).

### 3.3 Exploiting the acquired vulnerabilities in FASNis‐induced dormancy states

**Fig. 6:**
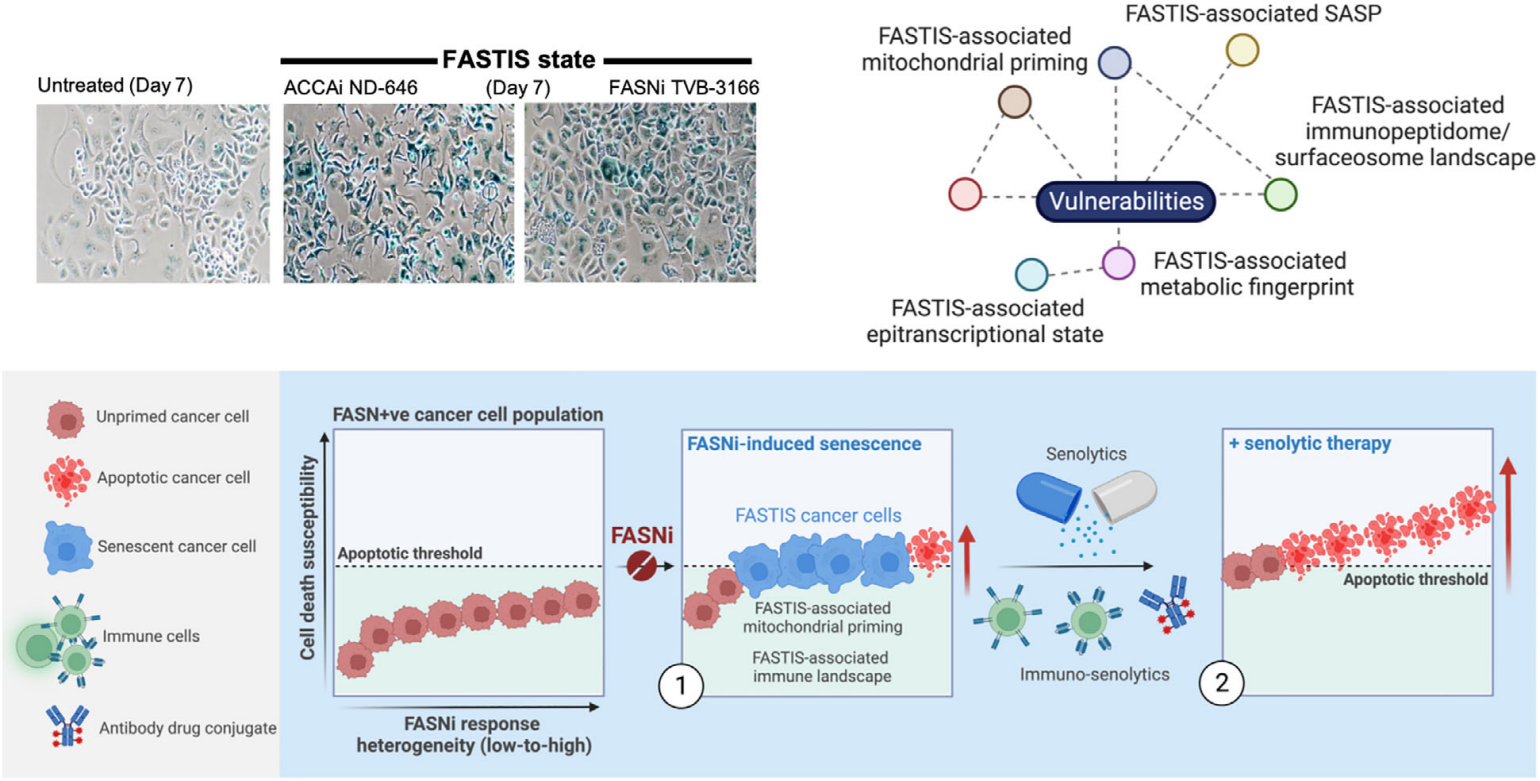
FA synthesis Therapy‐induced Senescence (FASTIS)‐based ‘one–two punch’ therapeutic approaches. FASN‐overexpressing cancer cells can persist after treatment with FA synthesis inhibitors by becoming senescent. Like other therapy‐induced senescence (TIS) states, the FASTIS state is expected to exhibit a concomitance of upstream pathways involved in resistance to cell death, and changes in transcriptional and epigenetic status, metabolic fingerprinting, secretome and immunogenicity due to altered expression of surface antigens and immune checkpoints. The identification of distinct, druggable vulnerabilities acquired by the FASTIS state can be exploited rationally to eradicate persistent cancer cells that survive FASNis, allowing the design of ‘one–two punch’ treatments using FASNis in combination with tailored senolytic drugs (e.g. BH3 mimetics) or immuno‐senotherapeutics targeting the FASTIS‐associated immune‐surfaceome. Created with Biorender.com.

A better understanding of the adaptive response of cancer cells to FASNis may provide new approaches to maximize their therapeutic benefit while preventing or delaying FASN‐driven disease relapse. Although the mechanisms by which cancer cells persist after treatment with FASNis remain largely unexplained, we have recently found that tumor cells can respond to FASNis by entering a dormant state. This is accompanied by the activation of several markers of senescence [209, 210, 211], including an enlarged and flattened cell shape, the upregulation of the cell cycle inhibitors p21 and p16, increased senescence‐associated β‐galatosidase (SA‐β‐gal) activity, and the activation of a senescence‐associated secretory phenotype (SASP) activity [212, 213, 214]. Since senescence activation in response to FASNis [214] was largely recapitulated in response to the clinical ACCA inhibitor ND‐646 [215] (unpublished observations), we have named this phenomenon FA synthesis Therapy‐induced Senescence (FASTIS). This adds lipogenesis inhibitors to the substantial number of mechanistically distinct anticancer interventions that induce senescence in cancer cells [216, 217, 218, 219, 220, 221, 222] (Fig. 6).

The senescence‐like cell cycle arrest that occurs after treatment with FASNis is not always permanent. It can be a form of tumor cell dormancy with an inherent potential to re‐emerge, generate progeny and contribute to disease recurrence [209, 210, 223]. Thus elimination of dormant tumor cells would provide a survival benefit for cancer patients. Indeed, the use of ‘clearing’ agents capable of eliminating residual FASTIS cells could maximize the therapeutic efficacy of FASNis – but how? Evasion of FASNi‐induced cell death in favor of senescence may come at a fitness cost of increased mitochondrial priming. For instance, mitochondria in FASTIS cells are expected to be primed by pro‐apoptotic BH3‐only proteins, creating an obligatory requirement for their sequestration to ensure survival. However, the types and levels of pro‐ and anti‐apoptotic factors that define the mitochondrial priming threshold to execute apoptosis in FASTIS cancer cells may differ from those that occur in proliferative cancer cells acutely treated with FASNis. Future studies should elucidate the adaptive dependencies on anti‐apoptotic proteins that sequester pro‐apoptotic molecules in the mitochondria to ensure the survival of FASNis‐induced senescent cells. The FASTIS state may also be accompanied by changes in chromatin structure, gene expression and metabolism (e.g. autophagy, proteostasis, redox maintenance, ferritinophagy), and cytokine production. FASTIS cells are expected to modulate immune responses by secreting SASP factors (i.e. FASTIS‐associated SASP) or by increasing the expression of specific surface antigens and/or immune checkpoints capable of recruiting/suppressing immune cells. FASTIS in cancer cells may therefore allow the design of a ‘one–two punch’ strategy, a type of sequential therapy that exploits the concept of collateral sensitivity [224, 225, 226, 227, 228, 229, 230, 231, 232]. The first therapy approach (FASNi) is not administered with curative intent, but rather to induce a metastable cellular state (senescence) that can be targeted with a second therapy (synthetic lethal) approach (e.g. senolytic drugs, immune response‐mediated senolysis) that selectively targets acquired vulnerabilities in FASTIS cancer cells (Fig. 6).

## 4 FASN: a multi‐faceted regulator of immune resistance

Targeting the immune checkpoints employed by cancer cells to evade immune recognition and destruction with immune checkpoint inhibitors (ICIs) has provided unprecedented clinical benefit in cancers with historically poor outcomes [233, 234, 235, 236, 237, 238, 239, 240, 241]. ICIs block the programmed cell death protein‐1 (PD‐1)/programmed cell death‐ligand 1 (PD‐L1) and cytotoxic T lymphocyte‐associated protein‐4 (CTLA‐4) immune checkpoint axes and reactivate the cytotoxic activity of exhausted T‐cells in the tumor microenvironment (TME). Deep tumor regression and remission have also been achieved with adoptive cell immunotherapies (ACIs), such as tumor‐infiltrating lymphocytes (TILs), natural killer (NK) cells, and chimeric antigen receptor (CAR)‐T and ‐NK cell therapies. These consist of reintroducing engineered immune cells that have been activated and expanded *in vitro* back into a patient [242, 243, 244, 245]. Unfortunately, only a minority of patients respond to the currently approved ICIs and ACIs, and the majority of patients are intrinsically refractory to immunotherapy and rapidly relapse after an initial response [246, 247, 248, 249].

FASN plays an immunosuppressive role in human tumors. On the one hand, FASN‐mediated *de novo* FA synthesis is critical for the functional maturation of regulatory T cells (T_reg_ cells). T_reg_ cells are essential for immune tolerance, but also drive immunosuppression in the TME [250]. Accordingly, loss of FASN from T_reg_ cells is sufficient to inhibit tumor growth. On the other hand, cancer cell‐intrinsic FASN prevents anti‐tumor immunity by reducing the ability of dendritic cells to maintain T‐cells in ovarian cancer [251] and is associated with reduced immune infiltration in gastric cancer [252]. Tumor FASN expression is part of an immune‐related signature that informs an immunosuppressive TME characteristic of immune‐deserted or ‐excluded tumors that may benefit from certain types of ICIs (i.e. anti‐CTLA‐4 blockade) in bladder cancer [253]. In melanoma and non‐small cell lung cancer (NSCLC) patients, FASN mutations that can inactivate FASN expression and functionality result in a favorable immune TME and improved response to ICIs [254]. Taken together, these findings strongly suggest that FASN blockade could help to overcome immune‐resistance and enable new therapeutic strategies to optimize immunotherapy in multiple ways.

### 4.1 FASN blockade: reversing immunoediting‐driven metabolic immunosuppression to overcome T‐cell dysfunction

FASN is part of the metabolic program directed by immunoediting to support immune evasion during initial tumorigenesis [255]. FASN is one of the many metabolic enzymes that tumor cells use to impede immune surveillance in coordination with immune‐related pathways, including PD‐L1 and CD47 expression [255, 256]. Importantly, loss of FASN in cancer cells impairs their ability to restrict CD8^+^ T‐cell infiltration and drive T‐cell dysfunction, strongly indicating that FASN blockade may suppress the ability of interferon‐gamma (IFNγ) to redirect tumor cell metabolism to support tumor immune evasion.

The strategy of priming cancer cells to become *softer* targets for cytotoxic immune cells via FASN suppression may be particularly relevant in immunologically ‘cold’ tumors. These tumors exhibit primary or acquired immune resistance due to various manifestations (or even the absence) of cancer immunosurveillance. These include immunoediting [e.g. major histocompatibility complex (MHC]) or antigen loss], activation of immunosuppressive pathways to dampen T‐cell reactivity (e.g. PD‐1/PD‐L1, CTLA‐4) and immune ignorance of ‘privileged’ antigens [257, 258, 259]. In the absence of strong antigens and no Darwinian‐like pressure from T‐cells (i.e. in the absence of immunoediting), tumor cells can remain susceptible to T‐cells, but only if the latter are primed or boosted. Indeed, insufficient T‐cell priming is now receiving increasing attention as a major cause of ‘cold tumors’ (lack of T‐cell infiltration) and their unresponsiveness to checkpoint inhibitors. The limited therapeutic efficacy of ACIs, which is largely dependent on the survival and replicative capacity of the implanted immune cells, may be due to a similar failure to mount cytotoxic responses to cancer cell antigens [260, 261, 262]. Once reintroduced into patients, engineered T‐, NK‐, CAR‐T‐ or CAR‐NK immune cells experience physiological levels of nutrients (e.g. glucose availability). These are far from the supra‐physiological levels used when they are activated and expanded *in vitro*, metabolically limiting their functionality and persistence *in vivo*. Consequently, the emerging field of cancer immunometabolism is largely devoted to understanding how tumor‐imposed metabolic constraints in the TME can impede the ‘nourished’ state of T‐cells. This field also focuses on how therapeutic approaches aimed at suppressing the metabolic quenching activity of the TME can enhance the differentiation, survival and effector functions of immune cells [263, 264, 265, 266, 267].

**Fig. 7:**
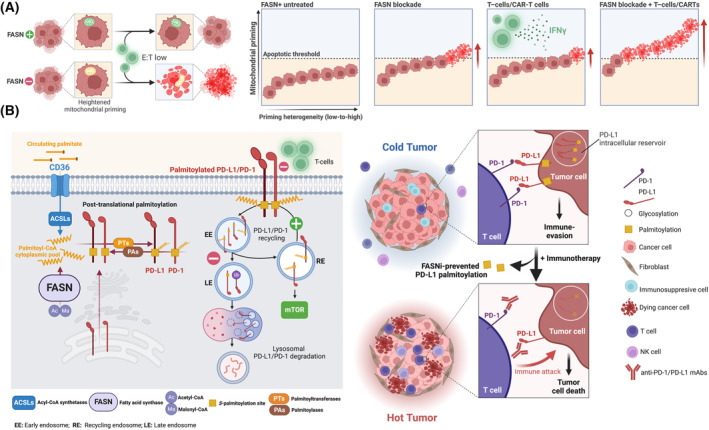
FASN and cancer immune‐resistance. FASN is a key component of the metabolic reprogramming that enables tumor cells to defeat T‐cell‐mediated immune surveillance. (A) Increasing cancer cell sensitivity to immune cells by targeting FASN. In physiologically relevant scenarios such as the tumor stroma, cytotoxic immune cells are almost always outnumbered by cancer cells. At these low effector (cytotoxic immune cell):target (cancer cell) ratios, the sublethal damage caused by a small number of E:T encounters could be amplified by mitochondria to push cancer cells over the apoptotic threshold. Whereas this mechanism may be unnecessary in ‘strong’, highly activated cytotoxic immune cells and/or high E:T ratios, it would be necessary in ‘weak’, dysfunctional/exhausted cytotoxic immune cells or when these cells are operating at low E:T ratios. Although FASN‐positive cancer cell populations are expected to be heterogeneous and vary from low to high mitochondrial priming, enhancing the mitochondrial apoptotic priming status with FASNis will bring the majority of tumor cells closer to the apoptotic threshold, thereby facilitating the cytolytic activity of immune cells. This mechanism may also operate to enhance the tumor‐intrinsic sensitivity to CAR‐T‐cell‐derived IFNγ that can occur at a distance without involving a direct contact, perforin‐mediated cytotoxicity [275]. (B) Exploiting the link between lipid metabolism and palmitoylation of immune checkpoints. Post‐translational palmitoylation of the immune checkpoints PD‐L1 and PD‐1 has a direct influence on their functionality and abundance. Although the palmitoylated status of these proteins is classically attributed to the catalytic activity of palmitoyl acyltransferases (PATs) and acyl‐thioesterases (ACTs), the bioavailability of exogenous and endogenous FAs is a key regulator of palmitoylation. Whereas an excess of exogenous FAs can be transported into cancer cells by CD36 and converted to palmitoyl‐CoA to enhance protein palmitoylation, FASN activity may ensure the synthesis of endogenous palmitate to replenish the cytoplasmic palmitoyl‐CoA pool and thus enable PD‐L1/PD‐1 palmitoylation independent of exogenous availability of FAs. Because FDA‐approved therapeutic antibodies competitively block PD‐L1/PD‐L1 on the cell surface, their benefits may be diminished by the palmitoylation‐dependent intracellular storage of PD‐L1/PD‐1 on recycling endosomes and their active redistribution to the cell membrane. Targeting PD‐L1/PD‐1 palmitoylation with FASNis could potentially exhaust the intracellular storage of PD‐L1/PD‐L1, leading to its depletion and allowing for more reliable and durable suppression. Created with Biorender.com.

An alternative but underexplored possibility may be to manipulate the intrinsic susceptibility of cancer cells to the cytolytic outcomes of the most important cells in tumor immune surveillance (T‐cells and NKs) via FASN suppression. FASN‐centered priming of cancer cells could also be considered to enhance the short lifespan and persistence of ACIs [268, 269, 270]. Contacts between cytotoxic immune cells and tumor cells are generally not binary ‘life/death’ events, but rather the damage done to cancer cells accumulates with multiple sublethal contacts with cytotoxic lymphocytes to reach a lethal threshold over time [271, 272, 273]. Importantly, mitochondria appear to be essential amplifiers of the death signal delivered by NK‐cells and cytotoxic T‐cells [274]. While a deficiency in mitochondrial apoptosis may render cancer cells resistant to killing by immune cells, increased mitochondrial priming may explain the additive cytotoxicity that occurs following serial encounters with cytotoxic lymphocytes. Since modulation of the priming status of mitochondrial apoptosis affects the susceptibility of cancer cells to T‐cells and NKs, priming of ‘low‐primed’ cancer cells could be used to bring them closer to the apoptotic threshold. This in turn could increase their sensitivity to subsequent cytotoxic contacts with immune cells (Fig. 7A). In this context, the ability of FASN signaling to dictate the mitochondrial primed‐for‐death state can be exploited to increase the vulnerability of cancer cells to immune cells. The increased mitochondrial priming induced by FASN blockade could bring cancer cells closer to the apoptotic threshold, thereby rendering ‘weak’ immune cells or less‐frequent hits due to low Effector (E; T‐cells):Target (T, cancer cells) ratios sufficiently robust to kill cancer cells. To test the hypothesis that FASN may control the intrinsic sensitivity of tumor cells to immune attack, we recently combined CRISPR/Cas9‐based gene knockout (KO) approaches in cancer cells with real‐time assessment of T‐cell‐mediated cytolysis. We found that a loss‐of‐function mutation in *FASN* in a near‐haploid (HAP1) cancer model dramatically enhanced T‐cell‐induced cytolysis and promoted a highly efficient killing kinetic of tumor cells by T‐cells even at low E:T ratios (unpublished observations). Tumor cell sensitivity to the pro‐apoptotic activity of IFNγ is an important determinant of CD4^+^ CAR‐T antitumor efficacy, even against antigen‐negative cancer variants [275]. Thus, the contribution of FASN to cancer cell death in response to IFNγ suggests that FASN may represent an interesting biomarker to identify patients who would benefit the most from a CAR‐T‐enriched infusion product. This may also suggest that FASN blockade may enhance the ability of CAR‐T cells to eliminate IFNγ‐sensitive tumor cells.

### 4.2 FASNi‐induced senescent cells: an opportunity for immune response‐mediated senolysis

There is increasing evidence that the SASP cytokines, chemokines and other factors secreted by senescent cancer cells can either promote or inhibit their immune clearance [224, 276, 277, 278, 279, 280, 281]. SASP factors can drive the recruitment of NK and CD8^+^ T‐cells to promote immune surveillance and eradicate senescent cells. Senescent cells can present senescence‐specific self‐peptides on an expanded MHC class I machinery to activate cytotoxic T‐cells and promote the eradication of senescent cancer cells. In contrast, some SASP cytokines can also attract immunosuppressive cells (e.g. myeloid‐derived suppressor cells, tumor‐associated macrophages, tumor‐associated neutrophils, cancer‐associated fibroblasts, regulatory T‐cells) to suppress the senolytic effects of NK cells and T‐cells [282, 283, 284, 285]. In addition, senescent cells can upregulate immune checkpoints such as PD‐L1 to attenuate T‐cell responses [286, 287]. These lines of evidence suggest that therapy‐induced senescence (TIS) may be exploited as a novel immunotherapeutic strategy [224, 281, 282, 283, 284, 285, 288, 289, 290]. If FASTIS cancer cells can stimulate the immune system to recognize and kill cancer cells, senescence induction as a mechanism of escape from FASNis could be exploited for the development of new immune response‐mediated senolysis approaches.

Oncogenic *KRAS* is associated with the induction of lipogenic gene signatures, establishing a specific dependency of *KRAS*‐mutant tumors on FASN activity [291, 292]. The clinical‐grade FASNi TVB‐2640, whose mechanism of action includes inhibition of KRAS palmitoylation and blockade of KRAS‐driven metabolic dependency, has shown clinical activity particularly in *KRAS*‐mutant lung, ovarian and breast cancers [53]. In *KRAS*‐mutant pancreatic and lung cancer models, senescence induction by trametinib and palbociclib enhances the direct senolytic effects of anti‐PD1 antibody therapy while improving drug delivery and immune cell recruitment via SASP‐facilitated vascular remodeling [293]. *KRAS*‐mutant models can therefore be used to explore whether the pro‐senescence effects of FASN blockade improve therapeutic outcomes when combined with immunotherapies. It should be investigated whether the epigenetic reprogramming that occurs during FASNi‐induced senescence leads to the re‐expression of germline‐encoded ligands on cancer cells (e.g. cancer/testis antigens). It is also worth examining whether FASNi‐induced senescence leads to the up‐regulation of antigen‐presenting genes (e.g. MHC class I molecules), and/or the generation of neo‐antigens [294, 295, 296, 297]. All of these phenomena may facilitate the synergistic recruitment and target recognition by activation of receptors on T‐cells. Moreover, the persistence of intratumoral senescent cells after therapy may be due to the upregulation of cell surface proteins that contribute to immune evasion. In this regard, a recent unbiased proteomic approach revealed that PD‐L2, but not PD‐L1, is upregulated in tumor cells after therapy‐induced damage and cellular senescence [298]. Furthermore, specific blockade of PD‐L2 could improve chemotherapy efficacy by reducing the intratumoral burden of senescent cells and the associated recruitment of immunosuppressive cells [298, 299, 300]. In our hands, the FASTIS state was accompanied by the transcriptional activation of PD‐L1 but not of PD‐L2 (unpublished observations). Thus, it will be important to determine whether cell‐surface vulnerabilities arising in FASTIS cancer cells are merely those broadly upregulated during senescence in normal cells (e.g. uPAR, B2M, DPP4, NOTCH1/3, etc.) [294, 295, 296, 297], or they are specific to FASNis in cancer cells (Fig. 6). For example, chimeric or antibody‐drug conjugated CART‐T cells can be engineered to specifically recognize and interact with these surface antigens present on FASTIS cells, promoting their clearance.

### 4.3 FASN‐driven protein palmitoylation: an understudied regulatory mechanism of immune checkpoint functionality

The post‐translational lipid modification of PD‐L1 and PD‐1 is a critical determinant of their stability, intracellular trafficking, subcellular localization and immunosuppressive capacity [301, 302, 303, 304, 305, 306]. PD‐L1 and PD‐1 palmitoylation is a process by which palmitate (16:0), is attached to the single palmitoylation sites Cys272 and Cys192 in the cytosolic domains of PD‐L1 and PD‐1, respectively [301, 302]. Palmitate (16:0) can either be taken up from the extracellular environment via CD36‐facilitated FA translocation or synthesized from non‐lipid nutrients (e.g. glucose and glutamine) via *de novo* lipogenesis [307]. PD‐L1 and PD‐1 palmitoylation prevents lysosome‐dependent degradation by suppressing ubiquitination or by promoting trafficking to the recycling endosome, ultimately increasing the cell surface distribution of PD‐1/PD‐L1 (Fig. 7B). This has been shown both by site‐specific point mutations using a cell‐penetrating competitive polypeptide encompassing the Cys272/Cys192 palmitoylation sites and by global inhibition of protein palmitoylation with the non‐metabolizable palmitate analog 2‐bromopalmitate (2‐BrP) [308, 309]. Palmitoylation of PD‐1, but not of PD‐L1, can also trigger mTOR signaling to promote tumor growth [302]. Disruption of PD‐L1/PD‐1 palmitoylation induces PD‐L1/PD‐1 lysosomal degradation, inhibits tumor cell proliferation and activates T‐cell cytotoxicity [301, 302, 310].

Preclinical studies have shown that targeting PD‐L1/PD‐1 palmitoylation may have promising immunotherapeutic effects [301, 302, 310], but there are currently no clinically testable approaches to target PD‐L1 palmitoylation. 2‐BrP, which blocks palmitoylation upon activation to 2‐bromopalmitoyl‐CoA or by blocking palmitate incorporation through direct covalent competition [308, 309], cannot discriminate among the broad portfolio of 23 cysteine‐rich Asp‐His‐His‐Cys (DHHC) domain‐containing palmitoyl acyltransferases. This will not provide the required specificity to target PD‐L1/PD‐1 palmitoylation in a cancer type‐dependent manner (e.g. DHHC9 catalyzes PD‐L1 palmitoylation in breast cancer, whereas DHHC3 specifically catalyzes PD‐L1 palmitoylation in colorectal cancer). It will also result in unwanted pleiotropic effects by suppressing palmitoylated proteins other than PD‐L1/PD‐1.

*De novo* synthesized palmitoyl‐CoA accumulates intracellularly in the range of 0.1–10 μmol·L^−1^ and serves as a palmitoylation donor. Although FASN actively contributes to the endogenous bioavailability of *de novo* synthesized palmitoyl‐CoA, the role of FASN as a regulator of protein acylation has been largely overlooked [311]. Pharmacological blockade of FASN has been shown to suppress the palmitoylation of several cancer‐related proteins such as EGFR, MYD88, AKT and the estrogen receptor, negatively affecting their stability, cellular localization, functionality and interactivity [312, 313, 314, 315, 316]. It is reasonable to speculate that FASN could modulate tumor immune evasion by altering the functionality of immune checkpoints such as PD‐L1 and PD‐1 via palmitoylation. As a proof‐of‐concept, we recently confirmed that CRISPR/Cas9‐driven KO of *FASN* completely abolishes PD‐L1 palmitoylation. This led to a significant reduction of PD‐L1 cell surface expression in a cancer cell model of constitutive PD‐L1 overexpression (unpublished observations).

## 5 FASN: a context‐dependent driver of metastasis

**Fig. 8:**
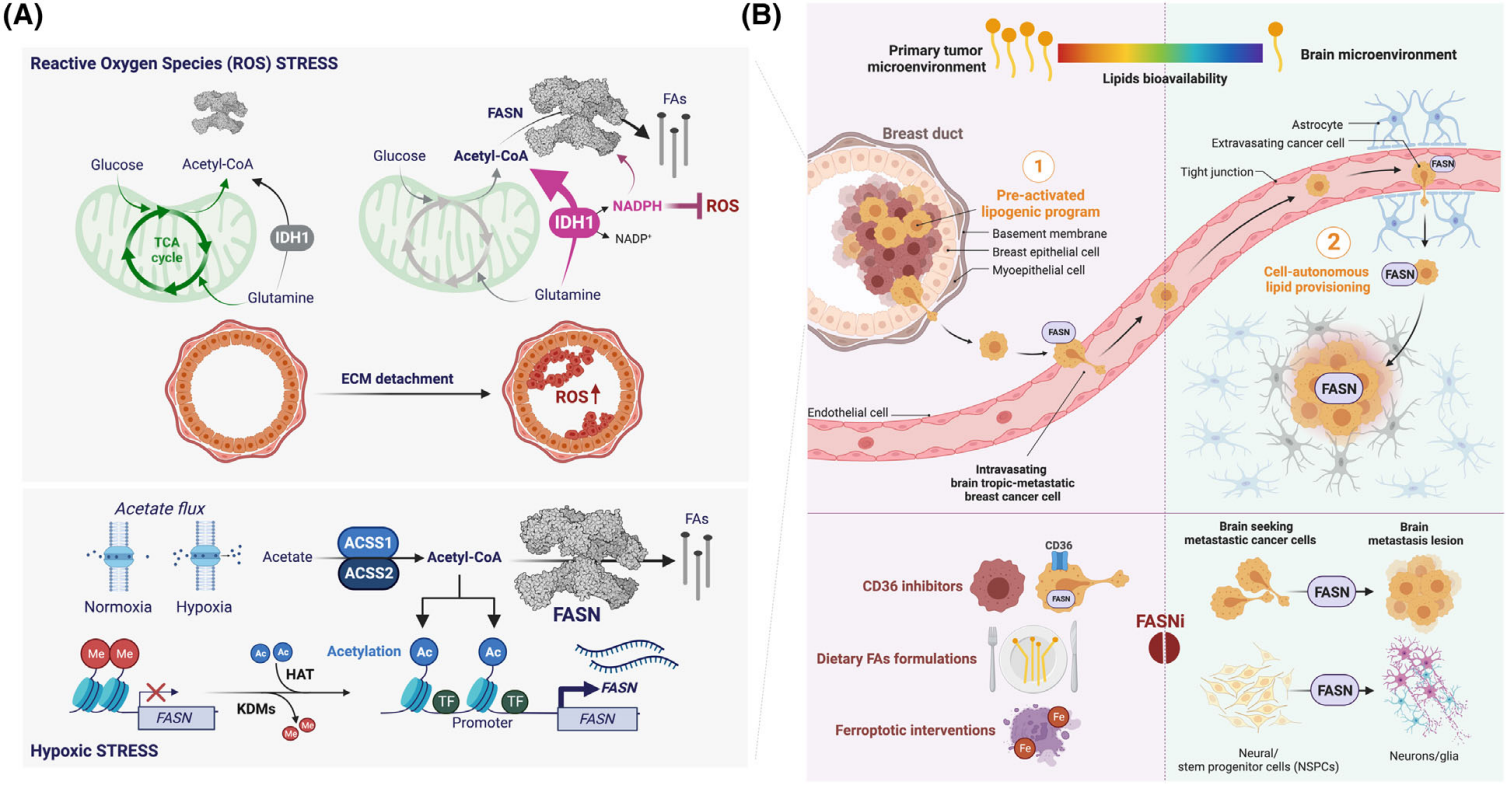
FASN‐driven metastasis: mechanisms and therapeutic opportunities. (A) FASN activation as a metabolic stress response to mediate anoikis and hypoxia resistance. *Top*. Upregulation of FASN in response to metabolic stress provides the necessary reductive power (via IDH‐dependent reductive carboxylation of glutamine) to tolerate the anoikis‐inducing oxidative conditions encountered by cancer cells when detached from the tumor matrix and during dissemination [23, 24]. *Bottom*. When the bioavailability of acetyl‐CoA from conventional carbon sources such as glucose and glutamine is limited under hypoxic conditions, the increased uptake of extracellular acetate allows its enzymatic activation to acetyl‐CoA, which in turn acts both as a metabolic precursor of FASN activity and as an epigenetic metabolite that activates the *FASN* gene promoter [317, 318, 319] (Ac, acetyl group; ACSS1/2, acyl‐CoA synthetase short chain family member 1/2; FAs, fatty acids; HATs, histone acetyltransferases; IDH1, isocitrate dehydrogenase 1; KDMs, histone lysine demethylases; TF, transcription factor). (B) FASN as a key component of the brain metastasis ‘dependency map’: Targetable metabolic vulnerabilities. *De novo* synthesis of FAs in the primary tumor and in the brain metastatic niche may facilitate the evasion and seeding of metastatic cancer cells as well as their escape from immune surveillance (*left*). Exacerbation of FASN expression and activity during the metastatic cascade offers distinct metabolic vulnerabilities that can be exploited to treat metastasis, namely: dual targeting of FASN and CD36‐driven lipid uptake in metastasis‐initiating cells; dietary interventions promoting reduced high‐fat exposure or specific dietary formulations that mismatch the availability of FA species with the lipidome of FASN‐inhibited tumors; and dietary and pharmacological interventions that hypersensitize cancer cells to FASNi‐induced ferroptosis. Lipid‐poor environments such as the brain will select brain‐tropic metastatic cells capable of cell‐autonomously generating FAs via upregulation of FASN (*right*). Although brain‐permeable versions of FASNis appear to be attractive candidates for targeting brain metastases, the proliferation, differentiation and survival of adult neural stem/progenitor cells (NSPCs) is also orchestrated by highly active FASN. Further studies should clarify the consequences of brain metastasis‐targeted FASNis on the ability of NSPCs to give rise to newborn neurons, astrocytes and oligodendrocytes in adult neurogenesis. Created with Biorender.com.

The highly complex phenomenon of metastasis requires metabolic, structural and signaling adaptations that allow cells to escape from the primary tumor to evade immune surveillance, and to migrate and colonize distant tissues. The multifaceted role of lipids in these processes involves the coordinated exploitation of major lipid metabolic pathways, including lipid uptake, catabolism, biosynthesis and lipid‐mediated cell death. The role of lipids, which ranges from the generation of metastasis‐initiating cells to metastatic outgrowth, has been reviewed elsewhere [10]. Tumor cells can also use lipid mediators and lipid modifications of proteins (e.g. palmitoylation) to overcome the many challenges along the metastatic cascade. In this context, FASN is gaining traction as a targetable metabolic node for metastatic cancer cells facing biophysical and nutritional constraints (e.g. oxidative stress, hypoxia, lack of exogenous palmitate) during early dissemination and distal colonization in hostile microenvironments (Fig. 8).

### 5.1 FASN: a lipogenic feature of early metastatic dissemination

By regulating their lipid metabolism, metastatic cells can better tolerate the oxidative conditions that occur during detachment from the tumor ECM and dissemination. Up‐regulation of FASN is part of the metabolic stress response used by tumor cells to achieve resistance or insensitivity to detachment‐induced cell death or anoikis [23, 24, 320]. This confers several advantages along the metastatic cascade [95, 321, 322, 323, 324, 325]. FASN activity, but not its biosynthetic end product palmitate, is an indispensable metabolic attribute to cope with the excessive ROS generated during the switch from 2D to 3D growth [23]. Specifically, the increased consumption of acetyl‐CoA by FASN unlocks the IDH‐dependent reductive carboxylation of glutamine to generate sufficient intra‐mitochondrial reducing equivalents necessary to quench ROS. In the absence of FASN, epithelial cells cannot undergo oncogenic malignant transformation *in vivo*, highlighting that the ROS buffering capacity of the FASN‐IDH axis is a critical checkpoint that governs the conversion of normal to malignant phenotypes. Increased *de novo* FA synthesis and regeneration of reducing power (NADPH) may be necessary to tolerate the metabolic changes that occur during metastatic dissemination (Fig. 8A). More specifically, these include the prevention of oxidative stress‐induced cell death, anoikis due to loss of epithelial interactions and a specific form of cell death known as ferroptosis (see below) due to circulation in an iron‐rich environment.

Metastatic cells may also epigenetically support FASN expression to promote cell motility and metastasis. The ability of cancer cells to produce acetyl‐CoA from conventional carbon sources such as glucose, glutamine and FAs is dramatically reduced under hypoxia [7, 317, 318, 319], an almost universal stress of solid tumors due to an inefficient vasculature. Hypoxic cells avidly take up acetate as a substitutive carbon supply to maintain the intracellular acetyl‐CoA pool under hypoxia. The conversion of acetate to acetyl‐CoA by acetyl‐CoA synthetases (ACSS1/ACSS2) provides a means by which acetate drives the lipogenic phenotype of hypoxic cells [317, 318] in two ways: as an immediate metabolic precursor of FASN activity and also by acting as an epigenetic metabolite to induce H3 activating acetylations (H3K9, H3K27 and H3K56) at the *FASN* gene promoter region [319]. Acetate‐driven activation of FASN could be viewed as an early metabolic‐epigenetic adaptation that enables post‐hypoxic tumor cells to acquire a ROS‐resistant FASN‐positive phenotype with enhanced survival to escape anoikis and to induce migration and invasion (Fig. 8A). Accordingly, FASN induction is associated with lymph node metastasis in cervical cancer and aggressiveness in liver cancers [326, 327, 328, 329, 330]. Preclinical studies suggest that FASN blockade reduces the clinical burden of melanoma and oral cancer cells by decreasing their angiogenic and invasive capacities [331, 332].

### 5.2 FASN: a lipogenic pre‐adaptation to colonize lipid‐deprived distal microenvironments

The availability of multiple nutrients such as glucose, pyruvate, glutamine, amino acids and FAs is a key aspect of a permissive environment that enables metastasis. Accordingly, cancer cells in distant organs should rewire their metabolism to survive on available nutrients [333, 334, 335, 336, 337, 338, 339, 340]. Although this is often viewed as an adaptive metabolic pressure that limits metastasis formation, it is also true that changes in the availability of some nutrients at the metastatic site can inherently promote metastatic growth. Interestingly, the FASN end product palmitate appears to be involved in both processes. On the one hand, lipid‐rich environments such as the lung – a common metastatic site for many tumors – may be promoted by pathological conditions such as pre‐metastatic niche formation and obesity to further increase the availability of palmitate [341]. Palmitate, in turn, promotes the expression of lysine acetyltransferases such as KAT2a, which will channel the available acetyl‐CoA to acetylate the nuclear factor kappa B (NF‐κB) subunit p65 and activate a pro‐metastatic transcriptional program in the lung. On the other hand, some lipid‐poor environments, such as the brain, may select for metastatic cells equipped with an enhanced capacity to endogenously biosynthesize *de novo* FAs in a cell‐autonomous manner. Indeed, two recent studies have shown that activation of lipogenic signatures involving FASN is a key metabolic adaptation hard‐wired into primary cancer lesions that can dictate patterns of brain‐specific distal colonization [31, 32] (Fig. 8B).

The so‐called ‘MetMap’, a novel strategy to define the molecular causes of organ‐specific metastatic patterns based on a compendium of 500 cell lines spanning 21 types of solid tumors, has recently shown that the brain metastatic potential of breast cancer cell lines is closely related to the presence of lipid‐synthesis traits [31]. A genome‐wide CRISPR/Cas9 viability screen identified the sterol regulatory element binding transcription factor 1 (SREBF1, also known as SREBP1) – the master regulator of the lipogenic gene transcription program including the *FASN* gene [342, 343, 344, 345] – as the strongest correlate of brain metastatic state. Thus, the enrichment for lipid‐metabolism signatures including SREBF1/SREBP1 can be characteristically observed in breast cancer cells in the brain and in patients’ brain metastases compared with other metastatic sites or their matched primary tumors [31]. Accordingly, brain metastatic lesions exhibit higher FASN‐driven *de novo* FA synthesis than tumors growing at other metastatic sites, a lipogenic adaptation that reflects exposure to the palmitate‐poor brain tissue environment [32]. Because the lipogenic characteristics of metastatic cells removed from the brain tissue are indistinguishable from those of the parental cells that initiate the tumors, it appears that the lipid‐poor brain environment actively selects for breast cancer cells pre‐equipped with a FASN‐positive lipogenic phenotype [32, 33, 34]. The requirement for FASN‐catalyzed *de novo* lipogenesis in brain metastasis is not shared with other common extracranial sites of breast cancer metastasis, such as the liver. This further emphasizes that the lipogenic activity of FASN is a brain site‐specific metabolic liability for metastatic breast cancer cells.

### 5.3 FASNis and brain metastasis: recognizing opportunities in challenges

FASN‐catalyzed endogenous FA synthesis is a novel driver of metastatic tissue tropism that allows breast cancer cells to adapt, survive and ‘proliferate’ in the brain [34]. In this context, medicinal chemistry‐based optimization of more brain‐permeable versions of FASNis may provide new opportunities to prevent and treat therapy‐resistant brain metastasis. As a proof‐of‐concept, CRISPRi‐mediated disruption of FASN expression was shown to efficiently impair the growth of breast cancer cells directly implanted in the brain or specifically extravasated and colonized the brain after intracarotid injection [32]. Central exposure to BI‐99179, a potent and selective FASNi with demonstrated peripheral and brain exposure after oral administration in animal models [72], was also found to significantly impair breast cancer cell growth in the brain, but not extracranially. However, it should be acknowledged that the problem of potentially severe side effects of targeting FASN at the brain level still remains (Fig. 8B). Similar to cancer cells, embryonic and adult neural stem/progenitor cells (NSPCs) upregulate the lipogenic pathway during proliferation [5]. Thus, pharmacological and genetic blockade of the high levels of FASN activity in proliferating NSPCs promotes a dramatic reduction in neurogenesis and directly impairs NSPC activity in learning and memory. Indeed, the quiescent versus proliferative behavioral status of NSPCs is under the tight control of Spot14, which acts as a regulator of FASN by reducing the levels of the FASN substrate malonyl‐CoA [346, 347, 348, 349, 350, 351]. Therefore, the development of next‐generation FASNis with active and homogeneous penetration into brain metastatic lesions should carefully consider how to circumvent the functional coupling between FASN‐dependent *de novo* lipogenesis and adult NSPC proliferation and function.

An interesting but still unexplored concept is whether FASN could be targeted for the prevention of brain metastatic disease as opposed to the treatment of established brain metastases. Whether activation of the lipogenic program is essential prior to brain seeding or is induced by the lipid‐poor brain environment remains to be determined. Although FASN overexpression alone may not predict the occurrence of brain metastases, activation of SREBF1/SREBP1 can predict which breast cancer cells have the predisposition to colonize the brain. This suggests that an extracranial selection for increased *de novo* FA biosynthesis may be a driver to promote the metastatic tropism of breast cancer to the brain [31]. If a particular subset of FASN‐positive cancer cells is unique in its ability to initiate brain metastasis, FASNis could act as a prophylactic to prevent and reduce the incidence of new brain metastases in high‐risk patients. In other words, the co‐option of FASN up‐regulation in metastatic cancer with organ‐specific tropism could be accompanied by therapeutically exploitable metabolic vulnerabilities. In this regard, we can consider at least three different FASN‐centered strategies to prevent early brain metastasis initiation/colonization [9, 335, 340, 352]: (1) dual blockade of lipid production/import, (2) dietary interventions and (3) promotion of lipid‐mediated cell death via ferroptosis (Fig. 8B).

#### 5.3.1 Co‐targeting FASN and lipid uptake

Metabolic plasticity can be a major obstacle to efficient targeting of metastasis‐specific metabolic vulnerabilities including FASN. The lipid requirements of tumors *in vivo* are met by the combined efforts of FASN‐driven *de novo* synthesis and lipid uptake, and therefore strategies that fail to target both pathways are likely to be less effective. Such lipogenic plasticity, i.e. the ability to salvage FAs from existing lipids as an alternative to endogenous FA synthesis, may be a critical mechanism of rapid adaptive resistance when incorporating FASNis into the clinical management of brain metastases. Reducing the availability of lipids by inhibiting lipid transporters could be considered as an adjuvant strategy to short‐circuit the ability of cancer cells to secure an alternative lipid supply in the presence of FASNis. Some, but not all FASNis, have the ability to activate compensatory exogenous lipid‐uptake mechanisms such as the scavenger receptor CD36 and *vice versa* [353, 354]. CD36 can bind and internalize long‐chain FAs, which can enhance the metastatic capacity of cells. Metastasis‐initiating cells are characterized by the presence of CD36, whereas blockade of lipid uptake by CD36 using chemical inhibitors or specific antibodies can inhibit metastasis formation or reduce the metastatic burden in animal cancer models [10, 334, 335, 339]. Therefore, combinations of FASNis with CD36 inhibition may lead to more effective anti‐brain metastasis strategies by forcing FASN‐positive metastatic cells simultaneously to overcome blocked lipid production and import. As a proof‐of‐concept, dual targeting of the cross‐talk between *de novo* lipogenesis and FA uptake has been shown to more potently inhibit the proliferation of human prostate cancer‐derived organoids when compared with single treatments [355]. FASN‐driven PD‐L1 palmitoylation may provide a novel mechanism by which metastatic cancer cells can overcome the innate and adaptive immune responses to enable brain metastasis colonization. This is due to the high concordance between primary and brain metastatic tumor PD‐L1 expression [356] and the association of PD‐L1 expression with early brain metastasis [357]. The potential role of FASN in dynamically regulating the palmitoylated or depalmitoylated state of CD36, which is known to abolish its FA uptake activity, deserves further analysis to provide a more complete understanding of FASN‐regulated palmitoylation as a preventive target for brain metastasis in combination with PD‐1/PD‐L1 blockade.

#### 5.3.2 FASNis and dietary interventions

Because the inhibition of FA synthesis can be compensated for by the uptake of circulating lipids, FASNis could significantly reduce tumor growth alone when combined with a low‐fat diet (LFD) [358]. Stearoyl‐CoA desaturase (SCD) is a lipogenic enzyme that synthesizes monounsaturated FAs and, similar to FASN, is required for cancer cells to proliferate in a lipid‐depleted environment. Diet‐induced mismatches between the availability of FA species and SCD ultimately determine whether low glycemic diets, i.e. calorie restriction (CR) versus a ketogenic diet (KD), can impair tumor growth [359]. CR leads to lower plasma and tumor lipid levels while inhibiting SCD activity, thereby creating an imbalance between unsaturated and saturated FAs to slow tumor growth. KD can also impair tumor SCD activity, but increases tumor lipid availability to maintain a balance between unsaturated and saturated FAs that allows tumor growth [359, 360]. The absence of FASN‐driven endogenous lipid production is not associated with a decrease in total FA content, but rather with an altered FA composition [359], demonstrating that the cancer cell lipidome that supports the development of FASN‐KO tumors is determined by dietary lipid composition. Therefore, the efficacy of FASNis as anti‐metastatic agents may be enhanced by LFDs or by interventions aimed at specifically altering the saturated/unsaturated ratios that enable tumor growth upon FASN blockade.

#### 5.3.3 FASNis and ferroptosis

Aggressive tumors with a FASN‐driven lipogenic phenotype show increased cellular levels of saturated FAs (SFAs) compared with non‐lipogenic tumors [22]. Pharmacological and genetic reversal of the lipogenic switch results in a marked decrease in saturated and monounsaturated FAs (SFAs and MUFAs) in cancer cells, while increasing the relative proportion of polyunsaturated FAs (PUFAs). Ferroptosis is an iron‐dependent form of non‐apoptotic, lipid‐mediated cell death triggered by lipid peroxidation of PUFAs resulting from an imbalance between ROS production from iron metabolism and antioxidant defenses [361, 362, 363, 364]. Since PUFAs, but not SFAs and MUFAs, are subject to lipid peroxidation, it seems intuitive to suggest that blockade of FASN‐driven lipogenesis may render cancer cells more susceptible to ferroptosis. This is the case in *KRAS*‐mutant lung cancers, where SREBP1/FASN‐driven *de novo* lipogenesis is used to limit the amount of PUFAs incorporated into membrane phospholipids, thereby averting lipid peroxidation and ferroptosis. KRAS‐driven cancers, but not wild‐type KRAS or EGFR‐mutants, appear to use endogenous FA biogenesis to survive the oxidative stress of some oxygen‐ and PUFA‐rich microenvironmental conditions, such as the lung [365, 366]. In this regard, TVB‐3664 has been reported to specifically induce ferroptosis in KRAS‐mutant lung cancer models [367]. This supports the notion that FASNis may act as ferroptosis‐inducing agents in cancer cells, using the lipogenic activity of FASN as a driver of the ferroptosis‐resistant cell state. Hypoxic and KRAS‐mutant cancer cells can escape ferroptosis by scavenging serum lysolipids to meet their demand for non‐peroxidable SFAs and MUFAs [368]. Similarly, the uptake of exogenous MUFAs to promote the displacement of PUFAs from plasma membrane phospholipids is a strategy employed by cancer cells in the lymphatic system to become less susceptible to ferroptosis prior to accessing the circulation [369, 370, 371, 372]. Activation of a FASN‐driven lipogenic state may circumvent the obligatory need of KRAS‐mutant, hypoxic and disseminated cancer cells to incorporate lipids actively as a protective mechanism against insults such as ROS‐dependent peroxidation of polyunsaturated acyl chains. Future studies should evaluate how FASN activity might dictate metabolic resistance to ferroptosis in iron‐enriched environments such as the blood, where metastatic cancer cells must regulate their membrane lipid composition to survive hematogenous dissemination.

Although seemingly counterintuitive, some studies have shown that FASN inactivation is part of the metabolic programs that promote resistance to ferroptosis [373, 374]. Expression of the antioxidant, lipid‐repair enzyme GPX4, which serves as a major ferroptosis suppressor under a variety of conditions [375, 376], is significantly attenuated in aggressive cancer cell types (e.g. triple‐negative breast cancer) with high levels of PUFAs and lipid peroxidation [377, 378]. Conversely, GPX4 is upregulated in mesenchymal therapy‐resistant cells as a defense mechanism against the increased levels of plasma membrane PUFAS that occur as a consequence of EMT [379, 380, 381]. Indeed, we now know that ferroptosis can be suppressed by both GPX4‐dependent and ‐independent mechanisms. Once one mechanism is inactivated or exhausted, cancer cells become dependent on the other mechanism to escape ferroptosis, while becoming highly sensitive to ferroptosis‐inducing conditions sensed by the original defense mechanism [382, 383]. Whether a GPX4‐related FASN on/off switch is similarly involved in the imbalance of the ferroptosis defense system depending on specific oncogenic mutations or specific cellular states requires further clarification. Since immunotherapy‐activated T‐cells enhance ferroptosis‐specific lipid peroxidation in tumor cells via IFNγ‐impaired uptake of the GPX4 promoter cystine [382, 384, 385], the involvement of FASN in prohibiting ferroptosis may also be explored for the development of pro‐ferroptotic therapy in the treatment of immune‐resistant, metastasis‐prone tumor cells. In addition, future studies should evaluate how FASTIS is associated with changes in the ferroptosis susceptibility of metastatic cancer cells, as senescent cells often accumulate and store intracellular iron as ferritin while limiting its autophagic degradation or ferritinophagy; however, excessive ferritinophagy can result in the release of large amounts of free iron, leading to lipid peroxidation and ferroptosis.

## 6 Conclusions

Metabolic inhibitors have been used in oncology for many decades, dating back to the folate antimetabolites introduced by Sidney Farber to combat leukemia in the 1940s. The discovery of strong links between oncogenes, tumor suppressors and metabolism that emerged in the 1990s has stimulated renewed interest in targeting the metabolic dysregulation of human cancer [386, 387]. As an example, the recent approval of small‐molecule inhibitors of mutant IDH in acute myeloid leukemia and cholangiocarcinoma [388, 389] represents a milestone in the precision targeting of cancer metabolism and supports the view that next‐generation metabolic therapy can be highly effective. However, the reality is that only a very limited number of anti‐cancer metabolic drugs have been developed in the last decade. Despite the breadth of cancer cell‐autonomous metabolic vulnerabilities targeted by an ever‐expanding portfolio of inhibitors, the most promising *in vitro* and preclinical studies typically do not translate into clinical success in early phase trials. This discrepancy between *bench* results and *bedside* effects has posed several challenges for the optimization of drug discovery in cancer metabolism.

**Fig. 9:**
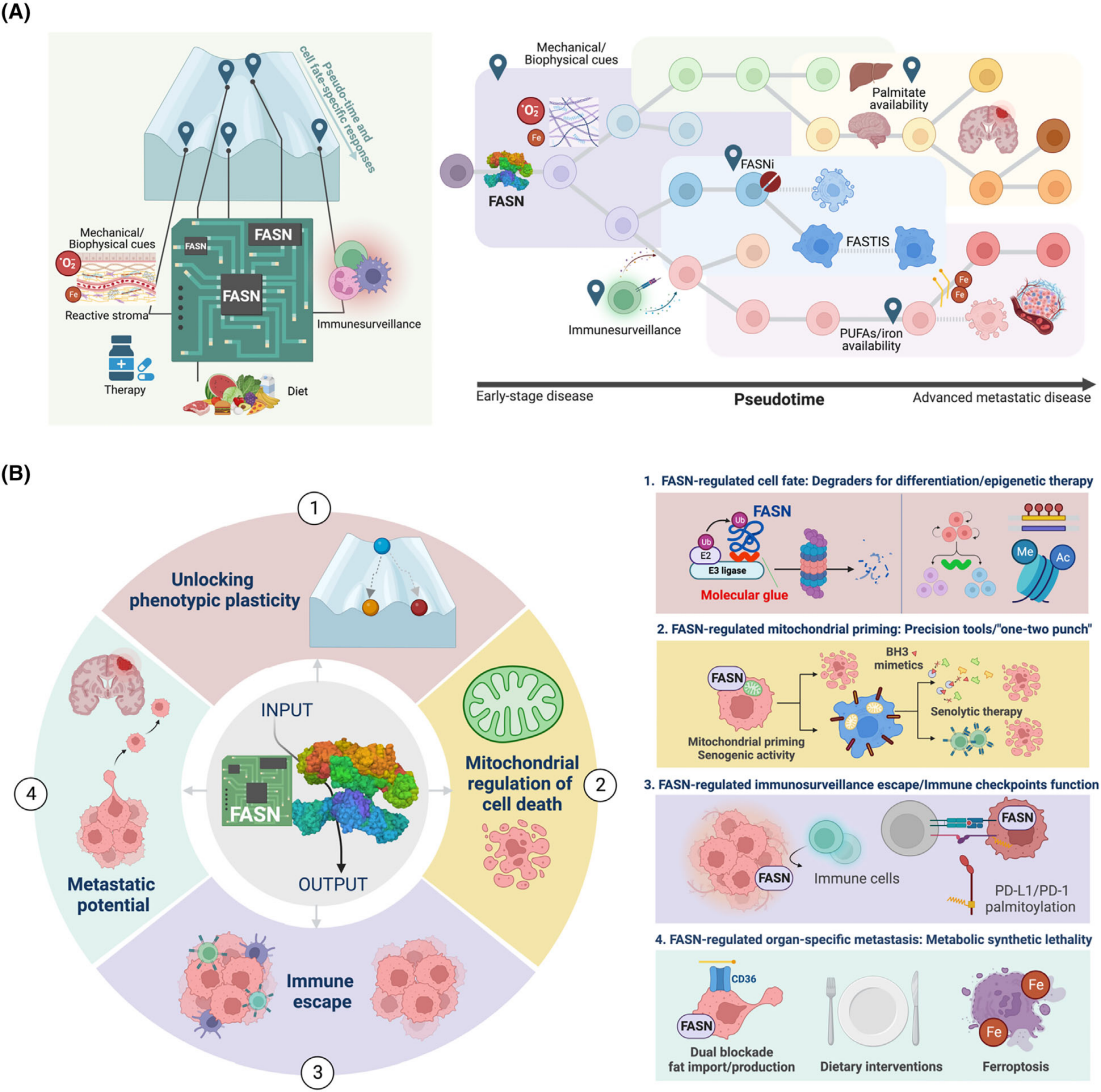
New dimensions of the FASN signalome in cancer: vulnerabilities and therapeutic opportunities. (A) Rethinking FASN signaling for functional precision oncology. FASN is an information processing system that mediates the communication between cancer cell intrinsic and microenvironmental cues to drive cancer pathophysiology from early tumorigenesis to metastatic disease. (B) The emerging hallmarks of FASN. First, we are now beginning to understand that the non‐catalytic functions of FASN mechanistically contribute to the cancer hallmark of ‘unlocking phenotypic plasticity’ by short‐circuiting various modes of cellular differentiation. Suppression of non‐catalytic FASN signaling will require the rational design of selective drug‐like PROTACs/molecular glues for targeted degradation of the FASN protein. Manipulation of the PPIs between FASN and its cognate ubiquitin E3 ligases may be a first step toward a new level of control over the non‐catalytic and catalytic activities of tumor FASN. Secondly, it has also become clear that the redox‐buffering activity of FASN is intrinsically linked to the mitochondrial apoptotic threshold in cancer cells. FASN blockade enhances mitochondrial apoptotic priming, which favors synthetic lethal interaction with direct activators of mitochondrial apoptosis, the so‐called BH3 mimetics, potentially unleashing the full therapeutic potential of FASNis. Adaptive resistance to FASNis may involve the acquisition of a senescent state with newly acquired therapeutic vulnerabilities. As such, FASNis are well‐suited to become part of a ‘one–two’ punch sequential cancer treatment with senogenic FASNis followed by senolytic therapy including BH3 mimetics and senescence‐targeting immunotherapeutics. Thirdly, one of the least explored areas of FASN signaling is its multifaceted ability to regulate cancer immune resistance. FASN inhibition can prime cancer cells for mitochondrial apoptosis to increase cancer‐cell susceptibility to immune cell‐mediated killing. The senogenic activity of FASNis may drive immune response‐mediated senolysis and synergize with checkpoint immunotherapy. FASN activity is a major contributor to the endogenous pool of palmitoyl‐CoA and therefore FASNis could be used to prevent the stabilization of post‐translational palmitoylation of immune checkpoints such as PD‐L1 and PD‐1. Fourthly, FASN activity is an obligatory lipogenic adaptation required not only to protect against oxidative stress protective during early metastatic dissemination, but also to overcome cell death in lipid‐deprived microenvironments (e.g. the brain) during late metastatic colonization and growth. The use of FASNis to increase the sensitivity of metastasis‐prone cells to specific dietary interventions and ferroptotic‐mediated cell death exemplifies the ever‐growing list of uncharacterized features of cancer‐associated FASN. Many of these FASN hallmarks are far removed from the anabolic, proliferative functions that were postulated for FASN when it was discovered as a metabolic alteration in most human tumors. Created with Biorender.com.

Reactivation of FASN‐driven *de novo* lipogenesis – the endogenous production of fats from simple metabolic precursors – is a hallmark of cancer that helps establish and maintain malignancy from early transformation to metastasis. To generate more accurate mechanistic models of the role of FASN in tumor initiation, progression and metastasis, we need to delineate an integrative understanding of FASN that extends beyond its historical status as a producer of endogenous FAs. Indeed, the concept of FASN as a fat megafactory in cancer cells is no longer applicable. The existing knowledge of FASN biology can be integrated under a common framework of signaling, in which FASN receives and integrates a repertoire of cancer cell intrinsic molecular signals and microenvironmental cues to drive responses during cancer initiation, therapeutic resistance and metastasis (Fig. 9A). Critical to this model is understanding how the highly complex FASN regulatory networks are coordinated to tune *FASN* mRNA and FASN protein levels according to the cancer cell needs of FASN activity. The molecular details of cancer‐associated FASN regulation are beyond the scope of the present perspective. However, it should be recognized that the long‐term and immediate effects of well‐established hormonal and dietary physiological regulators of FASN in normal tissues (e.g. insulin, glucagon, cyclic AMP, fructose, glucose, dietary fat) are poorly understood. [390, 391, 392, 393]. Perhaps more importantly, we are beginning to appreciate that prominent FASN activation or repression phenomena that occur in short time frames (e.g. minutes) involve a variety of post‐translational mechanisms. These include ubiquitination, acetylation, phosphorylation [394], SUMOylation [395], O‐GlcNAcylation [396, 397, 398, 399, 400], and likely allosteric regulation and protein–protein interactions. Future investigations are certainly needed to understand fully how post‐translational modifications allow cancer cells to fine‐tune FASN signaling in response to various microenvironmental and intracellular cues.

An in‐depth deconstruction of FASN as an input‐to‐output signal transmitter that transduces biological information to elicit cancer aggressiveness and lethality might inform molecularly guided strategies to optimize FASN and endogenous lipogenesis as metabolic targets for precision oncology. Although more than two decades have passed since the first description of FASN as a novel metabolic vulnerability for cancer prevention and treatment, progress in bringing FASN‐centered therapeutic strategies to the clinic has been slow. To date, most reviews of FASN in cancer have largely focused on the regulatory mechanisms underlying FASN overexpression in human cancers, its prognostic and clinicopathologic significance, and its association with biological features acquired during the multistep development of human malignancies [6, 7, 8, 9, 10, 16, 34, 35, 46, 60, 62, 63, 64, 65, 116, 386, 401, 402]. Here, our goal was to develop a new framework to overcome the current limitations in basic research and drug discovery in the targeting of FASN‐associated lipogenic metabolism in cancer cells, while at the same time providing translatable, personalized information to guide FASN‐targeted therapies. We carefully dissected the under‐studied involvement of non‐canonical and canonical FASN signaling in key aspects of cancer pathogenesis, such as phenotypic plasticity, cell death evasion, immune resistance and organ‐specific metastatic potential (Fig. 9B). Our current description of the vulnerabilities in the FASN signalome provides a roadmap for therapeutically directing FASN‐positive lipogenic cancer cells toward desired benign outcomes. In addition, it was intended to further our understanding of how the inhibition of FASN might counterintuitively promote the acquisition of aggressive features that are associated with cancer progression [403, 404]. These molecularly driven advances should certainly accelerate the optimization of FASN as a long‐awaited (almost 30 years!) metabolic target for precision oncology.
